# Influencing ionic conductivity and mechanical properties of ionic liquid polymer electrolytes by designing the chemical monomer structure

**DOI:** 10.1080/15685551.2023.2267235

**Published:** 2023-10-11

**Authors:** Lisa Ehrlich, Doris Pospiech, Petra Uhlmann, Felix Tzschöckell, Martin D. Hager, Brigitte Voit

**Affiliations:** a Department Polymer Structures, Leibniz-Institut für Polymerforschung Dresden e.V, Dresden, Germany; b Technische Universität Dresden, Organic Chemistry of Polymers, Dresden, Germany; c Institut für Organische Chemie und Makromolekulare Chemie (IOMC), Friedrich-Schiller-Universität, Jena, Germany

**Keywords:** Lonic liquids, chloride-conducting polymers, polymer electrolytes, polymer electrolyte networks, organic radical batteries

## Abstract

Polymeric single chloride-ion conductor networks based on acrylic imidazolium chloride ionic liquid monomers AACXImCYCl as reported previously are prepared. The chemical structure of the polymers is varied with respect to the acrylic substituents (alkyl spacer and alkyl substituent in the imidazolium ring). The networks are examined in detail with respect to the influence of the chemical structure on the resulting properties including thermal behavior, rheological behavior, swelling behavior, and ionic conductivity. The ionic conductivities increase (by two orders of magnitude from 10^−6^ to 10^−4^ S·cm^−1^ with increasing temperature), while the complex viscosities of the polymer networks decrease simultaneously. After swelling in water for 1 week the ionic conductivity reaches values of 10^−2^ S·cm^−1^. A clear influence of the spacer and the crosslinker content on the glass transition temperature was shown for the first time in these investigations. With increasing crosslinker content, the *T*_*g*_ values and the viscosities of the networks increase. With increasing spacer length, the *T*_*g*_ values decrease, but the viscosities increase with increasing temperature. The results reveal that the materials represent promising electrolytes for batteries, as proven by successful charging/discharging of a p(TEMPO-MA)/zinc battery over 350 cycles.

## 1. Introduction

Fast transformation to sustainable energy sources to mitigate climate change strongly requires efficient techniques for energy storage, for instance diverse types of batteries. Efficient batteries with high energy storage densities, on the other hand, need efficient systems in which all components (packaging system, current collectors, electrodes, electrolytes, and membranes) are optimized and work perfectly together [1]. This is still a challenging task and optimization needs to be done for all the different battery cells in use (*e.g.*, lithium-ion batteries, (lithium) metal batteries, and organic redox flow batteries) in a different way. However, the main tasks for all types include providing high charge carrier density, high carrier mobility, and fast carrier transfer through the different interfaces present in such a battery.

Since solid-state batteries are at the center of research with respect to safety reasons and performance, a particular focus has been set on solid-state electrolytes based on polymers. Development and optimization of these electrolytes can only be performed based on reliable structure–property relationships. As summarized in a number of recent reviews, much research has been done in the field of solid electrolytes for lithium ion [2–5]; lithium metal [6,7] and lithium-sulfur [8] batteries. All these reviews highlight the importance of polymer electrolytes for solid-state batteries. In most of the studies, gel polymer electrolytes (*i.e.*, polymers with conducting salt swollen in suitable solvents) or composite polymer electrolytes have been employed to enhance the only moderate ionic conductivity of ionic polymers which is caused by the type of ion transport [9,10]. The ionic conductivity of liquids is mainly determined by the viscosity, the concentration and charge of the ions, and the diffusivity, which is in turn a function of temperature (all correlations expressed in the Einstein, Stoke-Einstein and Maxwell equations) [10]. In contrast, ionic conductivity in polymers is primarily determined by the segmental mobility of the polymer backbone and proceeds *via* ion hopping along and across the polymer chains. This movement is by orders of magnitude slower compared to liquid electrolytes, but depends also on the diffusivity of the moving ions, *i.e.*, on the temperature. Despite this fact, the newer reports outline the importance of single ion conducting polymers as polymer electrolytes [6,7]. In addition to the research for metal and metal ion batteries, intensive activities focused on organic radical batteries [11–15] to avoid expensive and rare metals and to exploit high charging speeds, cycling stability, and discharging power capability of organic systems [16].

Polymers are the first choice for application in solid-state batteries due to their superior material properties such as flexibility (also in thin films), processability from solution and melt, as well as wide tunability of properties by their chemical structure and architecture. Polymeric materials can principally replace all components of batteries (electrodes, electrolytes and current collectors). While redox polymers have been demonstrated for use as negative and positive pole [16,17], the electrolyte task appears to be more complicated. The first studies employed liquid electrolytes that are standard for lithium-ion batteries [10,14] and, moreover, if possible with the electrodes used, also aqueous solutions of lithium *bis*(trifluoromethane sulfonyl)imide (LiTFSI), Mg(ClO_4_)_2_ or NaCl [16]. However, later on, the dissolution of certain polymer electrodes (for instance poly(TEMPO-methacrylate) p(TEMPO-MA)) in the electrolyte was observed [17]. Therefore, subsequent studies employed ionic liquids (such as Et_4_NTFSI) with dissolved conducting salt (mostly LiTFSI, but also pyridinium_14_TFSI) [17] as electrolytes, later on followed by the application of gel polymer electrolytes (IL plus conducting salt as swelling agent in a crosslinked polymer matrix) [18,19].

In this contribution, single chloride ion-conducting polymer electrolytes without the addition of swelling agents were studied to elaborate structure–property relations necessary for optimization of all-organic solid-state batteries. Polymer electrolytes contain structural elements of ionic liquids (ILs). Their synthesis and parts of their characterization were reported recently [20]. The selection of their chemical structure is based on former research on polymer electrolytes for lithium-ion and lithium batteries [21] with TFSI counterions. In that work, we could show that poly(vinyl imidazolium) polymers with TFSI counterions intensively examined before [22–24] achieved ionic conductivities in the range of 10^−8^ S⋅cm^1^ at 20°C. However, this range is not high enough to provide efficient electrolytes (target: 10^−4^ S·cm^−1^). Moving to poly(acrylate)s with substituents having alkyl spacers linked to imidazolium rings enhanced the ionic conductivities to target the range of 10^6^ to 10^−4^ S.cm^1^ [21]. This basic structure was then employed for electrolytes with chloride counter ions [20]. Despite rather high ionic conductivities, the performance of battery cells with p(TEMPO-MA)/Zn electrodes with these polymer electrolytes was not really satisfying. The following reasons were considered: still insufficient ionic conductivity, heterogeneous morphology of the crosslinked electrolyte with conducting salt, and insufficient ion transfer at the electrode surfaces. Furthermore, it was shown for selected samples that the ionic conductivity of the electrolyte systems could be increased by two orders of magnitude with increasing temperature (from 20°C to 70°C). Thus, it was concluded that further research for optimization, in particular with respect to structure–property relations, is required for open applications in organic polymer batteries.

Therefore, in this study, the influence of the chemical structure of the base IL monomers on the properties of linear homopolymers as well as polymer networks was examined in more detail, including thermal behavior (glass transition temperature), swelling behavior, rheology and the relation to ionic conductivity in dependence of temperature. This research shall gain basic knowledge necessary to efficiently transfer the materials into working battery components.

## 2. Experimental

### 2.1. Materials

Acryloyl chloride (96%, Alfa Aesar), 3-chloro-1-propanol (98%, Sigma Aldrich), 6-chloro-1-hexanol (96%, TCI), 8-chloro-1-octanol (98%, Sigma Aldrich), 1-butylimidazole (99%, abcr), 1-hexylimidazole (>98%, IoLiTec), tetrahydrofuran (THF, 99.5%, Acros Organics, stored over molecular sieve 0.3 nm), triethylamine (≥99.5%, TEA, Sigma Aldrich), diethyl ether (99.5%, ChemSolute), ethyl acetate (≥99.8%, fisher scientific), magnesium sulfate (anhydrous, Alfa Aesar) were used for the synthesis and purification of the monomers. The non-ionic crosslinker *N,N’*-diethyl-1,3-bis(acrylamido)propane (BAAP) was provided by Ivoclar Vivadent AG (Schaan, FL). The photoinitiator diphenyl(2,4,6-trimethylbenzoyl)phosphine oxide (97%, TPO, Sigma Aldrich), the conducting salts tetrabutylammonium chloride (TBACl, 98%, TCI), zinc perchlorate hexahydrate (99.997%, Thermo Fisher Scientific), and 1-hexyl-3-methylimidazolium chloride (>98%, IoLiTec) were used as received. Milli-Q water was used as a swelling agent. Zinc electrodes (99.995%, thickness 0.1 mm, Alfa Aesar), separator Celgard 2500 (thickness 25 µm, CELGARD, North Carolina, U.S.A.), ethylene carbonate (anhydrous, ≥99%, EC, Sigma Aldrich), dimethyl carbonate (anhydrous, 99%, DMC, Sigma Aldrich) and p(TEMPO-methacrylate) (p(TEMPO-MA)) electrodes were prepared in the Jena group according to former work [25].

### 2.2. Preparation of IL monomers and PIL networks

#### 2.2.1. Acrylic imidazolium chloride IL monomers AACXImCycl

**Figure 1.**
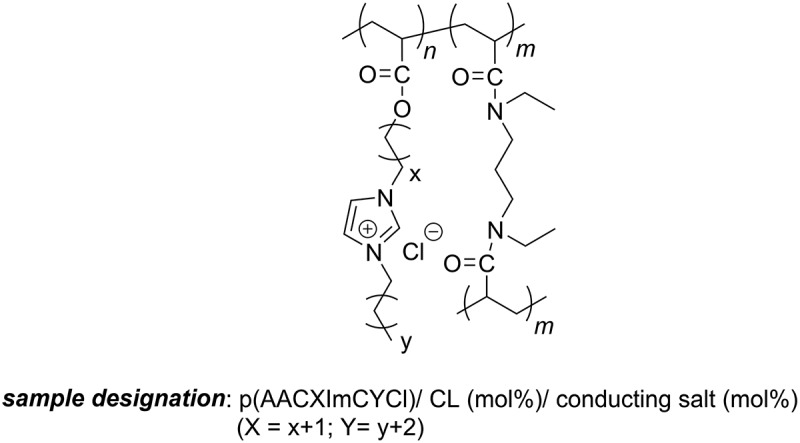
Chemical structure of the polymer electrolytes examined in this study (*m* = 0: linear homopolymers; *m* > 0: polymer networks).

The synthesis of the ionic liquid monomers AAC3ImC4Cl, AAC6ImC4Cl, AAC6ImC6Cl, AAC8ImC4Cl (compare Figure 1) was performed in a two-step reaction and the purity of the monomers was determined with NMR as reported in the work of Ehrlich *et al*. [20]

#### 2.2.2. Preparation of IL network films

A mixture of the IL monomer, photoinitiator TPO (1 mol% with respect to the IL monomer used), different amounts of crosslinker BAAP (0–10 mol% with respect to the monomer), and conducting salt TBACl (0–5 mol% with respect to monomer) was prepared and stirred in the dark at 30°C for about 2 h. After complete dissolution, the mixture was degassed under argon and transferred into a glovebox. The mixture was casted into a Teflon mold for DSC measurements (diameter: 10 mm; height: 100 or 250 µm), rheology plates (diameter: 8 mm) or Swagelok cells (diameter: 10 mm) and was polymerized by UV light (365 nm), Herolab GmbH for 30 min (*in situ* polymerization).

### 2.3. Battery cells

The polymer electrolyte was synthesized as described in section 2.2.2 with monomer AAC6ImC4Cl, 1 mol% TPO, 5 mol% crosslinker, 5 mol% zinc perchlorate hexahydrate as conducting salt and IL 1-hexyl-3-methylimidazolium chloride. The amount of ionic liquid was calculated from the amount of monomer: vol_monomer_/vol_IL_ 1/2. After transfer into a glovebox, the electrolyte mixture was casted directly on the p(TEMPO-MA) cathode (diameter 16.4 mm) and were diffused into the active material for 1 hour under reduced light conditions. Afterward, the monomer mixture was polymerized on the electrode for 30 min and then installed in the coin cell with zinc (16.4 mm) as the anode.

### 2.4. Measurements

#### 2.4.1. Nuclear magnetic resonance (NMR) spectroscopy

NMR was performed on an Avance III 500 Spectrometer (Bruker, Germany) at ambient temperature (^1^H NMR: 500.13 MHz, ^13^C NMR: 125.74 MHz). Dimethyl sulfoxide (DMSO-d6) was used as solvent for all samples *(δ*(^1^H) = 2.50 ppm, *δ*(^13^C) = 39.6 ppm).

#### 2.4.2. Differential scanning calorimetry (DSC)

DSC measurements were performed on a DSC Q5000 (TA Instruments, Newcastle, DE, U.S.A.) under nitrogen. After 2 days drying in the glovebox, the samples were dried at 45°C in a vacuum oven for 24 h and stored in a desiccator before the measurement started. Samples were subjected to heating-cooling-heating cycles in the temperature range from −90 to 150°C with scan rates of 10 K min^−1^. The glass transition temperatures were determined from the first heating run.

#### 2.4.3. Rheology

Viscosity measurements were performed on an ARES rheometer (Rheometric Scientific, RHEO Service GmbH & Co. KG, Reichelsheim, Germany) with a Force Rebalance Transducer (FRT) and a parallel-plate geometry in a dynamic temperature ramp test. The samples were prepared directly on the rheology plate with a diameter of 8 mm, 1 day before the measurement was performed. Samples were subjected in the temperature range from 20°C to 80°C with scan rates of 5 K min^−1^, a frequency of 5 rad s^−1^ and a strain of 1%.

#### 2.4.4. Electrochemical impedance spectroscopy (EIS)

EIS measurements were performed in Swagelok cells (Swagelok Co., Solon, OH, U.S.A.) with a symmetrical cell setup and stainless steel (SS) electrodes (SS/PEL/SS). The polymer films were prepared as described in section 2.2.2 directly on the one side of the Swagelok cell in the glove box (Sylatech GmbH, Walzbachtal, Germany). The thicknesses of the polymer films were determined by a digital calliper gauge device (Carl Roth GmbH + Co. KG, Karlsruhe, Germany). The measurements were carried out with a potentiostat (BioLogic, Seyssinet-Pariset, France) with the following parameters: 1 MHz to 10 Hz at open-circuit voltage of 10 mV, AC current and heated in a temperature chamber (BINDER GmbH, Tuttlingen, Germany) from 20°C to 80°C in 10 K steps with an equilibration time of 1 h for each temperature. The ionic conductivity *σ*_*ion*_ is calculated by Equation (1)

(1) $$\sigma_{ion}=\frac{d}{A*R_{b}}$$

where *d* is the sample thickness, *A* the cross-sectional area of the sample (0.785 cm^2^) and *R*_*b*_ is the bulk resistance of the sample, determined by the Nyquist plot.

#### 2.4.5. Swelling behavior of the networks

The swelling tests were used to determine the mass-related average degree of swelling as a function of the crosslinker and conducting salt content of selected samples at room temperature (22.4°C). Three independent samples of the same batch were swollen in Milli-Q water for 7 days. The swollen samples were then removed from the swelling medium, the interstitial water was eliminated with filter paper and weighed again to determine the mass-related degree of swelling.

#### 2.4.6. Scanning electron microscope (SEM)

The SEM measurements were performed on a Gemini Ultra Plus microscope (ZEISS, Germany) with a SE2 detector with an aperture size of 30 µm and a canon vacuum of *6.6·10*^*-10*^ mbar and a system vacuum of *2.5·10*^*5*^ mbar. For this purpose, the samples were first cast in epoxy resin and cured for 24 h. Before measurements, the samples were glued to copper tape and sputtered with 3 nm platinum.

#### 2.4.7. Galvanostatic cycling test

The battery tests were performed on a Battery Analyzer BST8-MA-220 (MTI Corporation, Richmond, California, U.S.A.). The cells were charged/discharged with a constant current (0.1C) at room temperature (22.4°C) in the voltage range from 0.5 V to 2.5 V for 350 cycles.

## 3. Results and discussion

The polymer electrolyte networks are composed of acrylic imidazolium chloride ionic liquid monomer AACXImCYCl, as reported previously [20]. Crosslinking was achieved under radical UV polymerization conditions by employing the acrylamide crosslinker *N,N’*-diethyl-1,3-bis(acrylamido)propane (assigned here to ‘crosslinker’ (CL)). In the previous studies, we investigated the UV polymerization using *in situ* RAMAN spectroscopy and detected complete conversion within a few minutes [20]. The chemical structure of the polymers is depicted in Figure 1.

This basic structure can meet several major requirements for polymer electrolytes with sufficient ionic conductivity. First, a certain decoupling of the segmental movement of the backbone and the counter ions should occur [22], second, the glass transition temperatures are well below ambient temperature to offer high segmental mobility [20], and third, most probably a nanophase separation between the unpolar backbones and the polar substituents may be generated to form tunnels, in which the ions can be transported. But this aspect will be examined in a further study. Moreover, the exchange of large TFSI^−^ ions (0.326 nm [26]) by Cl^−^ ions in the polymers studied reduces the size of the ions to 0.181 nm [27], which should also support a faster movement of ions, and thus, result in higher ionic conductivity.

If m in the polymer structure illustrated in Figure 1 is zero, linear homopolymers are obtained, and in the case of *m* > 0, polymer networks are obtained. The molar amount of crosslinker was varied between 0, 2.5, 5 and 10 mol% to figure out the influence of the crosslinking density. The alkylene spacer had either three (X = 3), six (X = 6) or eight (X = 8) methylene groups, while the substituent in the imidazolium group was varied between four (Y = 4) and six (Y = 6).

In the following, the temperature dependence of different properties of these polymers was investigated and the correlation to the number of alkylene groups was revealed. Maintaining preparation, drying, and storage conditions comparable for all samples should provide reliable conditions to find such correlations.

### 3.1. Thermal behavior of the IL polymer electrolyte networks

The glass transition temperatures *T*_*g*_ of the synthesized polymers were investigated by DSC using the first heating run. Usually, the 2^nd^ heating is employed for the determination of *T*_*g*_ to avoid the influence of the thermal sample history. In contrast, the first heating was chosen here, since all further measurements (complex viscosity, EIS, swelling and finally also application as polymer electrolytes in battery cells) will take place under such conditions. Thus, the first heating run reflects the behavior of the PELs under these conditions better than the second heating.

In the 1^st^ heating run, no further thermal effects of the samples occurred. The DSC curves of all samples are provided in the Supporting Information (SI; Figure S1 – Figure S4). Because of the high sensitivity of the DSC measurements towards moisture, all samples were dried and stored by the same procedure as described in the Experimental Section.

**Figure 2.**
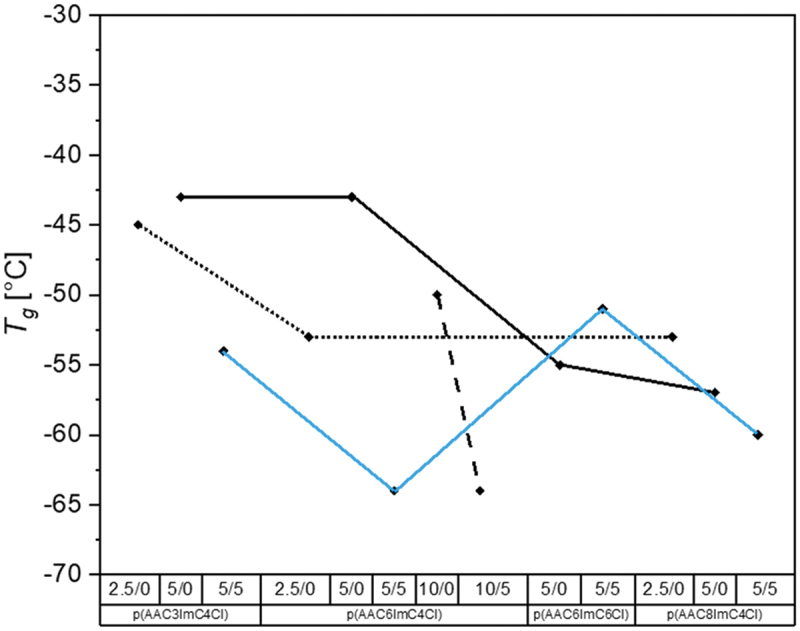
Glass transition temperatures of all investigated PIL networks with varied alkyl spacer X and imidazolium substituent lengths Y as well as crosslinker and conducting salt content (p(AACXImCYCl)/CL (mol%)/conducting salt (mol%)).

Keeping the segmental mobility of the polymer chains at room temperature as high as possible is important for the application as solid-state electrolyte in batteries to allow ion hopping from segment to segment. Therefore, *T*_*g*_ values below 0°C are desired. For this purpose, the variation of alkyl spacer, alkyl substituent, concentration of crosslinker and conducting salt tetrabutylammonium chloride (TBACl) was performed to understand the influence of the polymer structure on the *T*_*g*_ values. These influences remained unclear in the previous studies [20] but can support the optimization towards PEL with improved ionic conductivity. The glass transition temperatures of all investigated polymer samples are summarized in Table 1 and are graphically shown in Figure 2. The values varied between −30°C and −65°C, depending on the polymer network structure.

**Table 1.** Glass transition temperatures of the investigated polymer network samples p(AACXImCYCl)/CL (mol%)/conducting salt (mol%).

PIL network	*T*_*g*_ [°C]
p(AAC3ImC4Cl)/2.5/0	−45
p(AAC3ImC4Cl)/5/0	−43
p(AAC3ImC4Cl)/5/5	−54
p(AAC6ImC4Cl)/2.5/0	−53
p(AAC6ImC4Cl)/5/0	−43
p(AAC6ImC4Cl)/5/5	−64
p(AAC6ImC4Cl)/10/0	−50
p(AAC6ImC4Cl)/10/5	−64
p(AAC6ImC6Cl)/5/0	−55
p(AAC6ImC6Cl)/5/5	−51
p(AAC8ImC4Cl)/2.5/0	−53
p(AAC8ImC4Cl)/5/0	−57
p(AAC8ImC4Cl)/5/5	−60

It was expected that the glass transition temperatures would generally be lower with increasing alkyl chain length as well as lower crosslinker content due to the enhanced flexibility of the chains and an increasing molar volume of the segments between network nodes [22,28]. Taking samples with comparable crosslinker content into account, this tendency could roughly be observed (for 2.5 and 5 mol% crosslinker), in contrast to former results with TFSI^−^ [21] and Cl^−^ [20] counter ions.

The *T*_*g*_ values with conducting salt (5 mol%) were in most cases (except p(AAC6ImC6Cl)) lower than those without. It can be reasoned that tetrabutylammonium chloride at low concentrations shows a strong interaction with imidazolium cations, an increase in free volume and, moreover, acts as plasticizer [28]. Previous studies have shown that a higher concentration of conducting salt (>5 mol%) had a negative effect on the performance of the networks due to the limited solubility of the salt in the monomer mixture [20]. According to the literature, larger counterions (TFSI^−^, PF_6_^−^, Tf_2_N^−^) interact stronger with imidazolium cations than smaller ones (Cl^−^, Br^−^, BF_4_^−^) giving a plasticizing effect. This is visible in reduced *T*_*g*_ [28]. In almost all cases (except sample p(AAC8ImC4Cl)/2.5/0) the lower amount of crosslinker (2.5 mol%) led to lower *T*_*g*_ values compared to the samples with 5 mol% crosslinker. The lowest values in ascending order according to the total alkyl number *N* = X+Y in the networks were with the monomers AAC8ImC4Cl (*N* = 12) < AAC6ImC6Cl (*N* = 12) < AAC6ImC4Cl (*N* = 10) < AAC3ImC4Cl (*N* = 7).

The deviating results of the samples p(AAC8ImC4Cl)/2.5/0 compared to p(AAC8ImC4Cl)/5/0 could be explained by the fact that very low amounts of by-products in the crosslinked polymer acted as plasticizers and the variation of crosslinker is too low for clear differences between them [20].

Summarizing the DSC results it is noted that correlations between the chemical structure of the monomers, the crosslinker content and the resulting *T*_*g*_s could be observed.

### 3.2. Viscosity as a function of temperature

**Figure 3.**
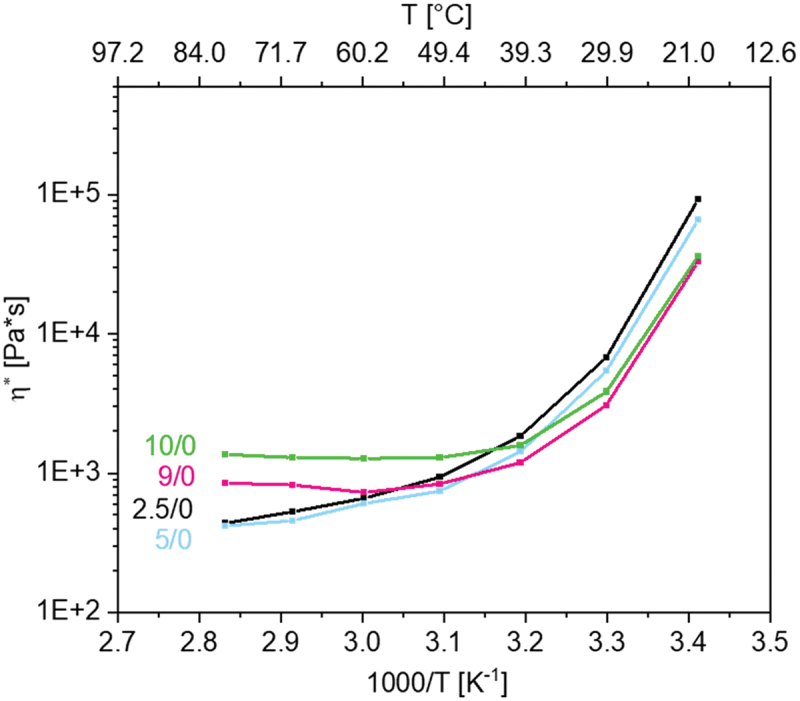
Complex viscosity of the p(AAC6ImC4Cl)/CL (mol%)/conducting salt (mol%) IL networks.

Besides the glass transition temperatures, viscosity is also one of the most important factors to understand the charge transport within polymer electrolytes. The ionic conductivity depends on the concentration of free ions, the charge of the ions and diffusivity [10]. In polymers, diffusivity is controlled by the segmental mobility. Thus, rheological measurements can give an insight into the influences on ionic conductivity. The viscosities of the IL networks were studied as a function of temperature in the range from 293.15 to 353.15 K. A temperature range above the *T*_*g*_ values was selected, since battery cells also operate in this range. Figure 3 shows the complex viscosities of networks p(AAC6ImC4Cl) with crosslinker contents varied from 2.5 to 10 mol% as a function of temperature.

In the temperature range from 293.15 to 323.15 K, the complex viscosities decreased strongly with temperature to a significantly reduced level. Starting at about 50°C, in some samples (in particular with higher CL contents) a plateau was reached. Then, the complex viscosities remain almost constant.

As illustrated in Figure 3, small variations in crosslinker content had only a weak effect on the viscosity of the networks at room temperature. Surprisingly, the networks with crosslinker content <5 mol% showed minimally higher viscosity values than those with >5 mol% CL. At higher temperatures, this phenomenon reversed, as assumed, and the expected tendency in the network behavior (higher viscosity with higher network density) occurred. At 80°C, the networks with 10 mol% CL showed the highest viscosity and those with <5 mol% showed significantly lower values.

**Figure 4.**
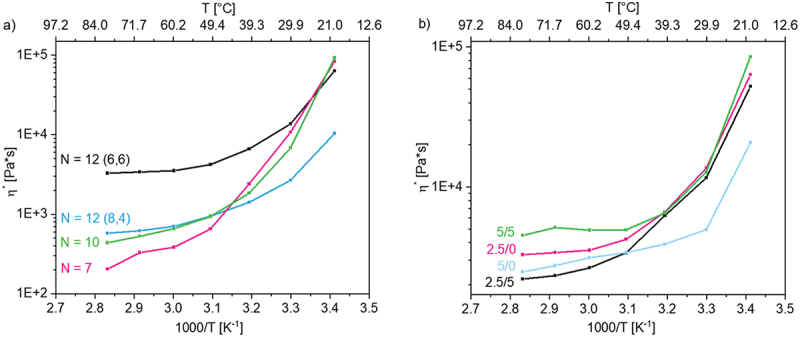
(a) influence of alkyl number N (total number of C atoms) with 2.5 mol% crosslinker and without conducting salt, and (b) influence of conducting salt and crosslinker on the viscosity in dependence of temperature of p(AAC6ImC6Cl)/CL (mol%)/conducting salt (mol%) network samples.

Not only the amount of crosslinker but also the influence of spacer and *N*-alkyl substituent as well as the amount of the conducting salt was examined in detail. Figure 4a shows the influence of the alkyl number *N* = X + Y (according to Figure 1) and the influence of the conducting salt tetrabutylammonium chloride (TBACl) (Figure 4b) for selected samples.

It can be recognized that the total number of C atoms (in alkyl spacer X plus *N*-alkyl substituent Y) plays a decisive role for the viscosity, i.e., the mechanical strength of the networks. As the number of alkyl groups increased, the viscosity of the networks at 80°C also increased. This behavior could not be observed at room temperature. The network p(AAC3ImC4Cl)/2.5/0 with X + Y = 7 showed the strongest change in viscosity over the temperature range examined. Possibly the crosslinker content of 2.5 mol% may not be high enough to form a stable network, which would explain the further decrease in viscosity at 80°C caused by mechanical destruction. A high stability is shown by the networks with X + Y = 12, where the length of the *N*-alkyl substituent had a strong impact. Thus, the viscosity of p(AAC6ImC6Cl)/2.5/0 (X + Y = 12(6,6)) was significantly higher compared to p(AAC8ImC4Cl)/2.5/0 (X + Y = 12(8,4)). However, the marginal decrease in viscosity (by around 1 order of magnitude) over the temperature range, compared to the other samples, and the plateau setting can be taken as evidence of a very stable system. Furthermore, it has to be taken into account that small amounts of remaining monomer act as plasticizer which may also alter the viscosity results slightly.

The amount of conducting salt also had a significant influence. In principle, conducting salt is used to increase the ion density, as charge carriers are introduced into the system. An increase in viscosity is expected after the addition of conducting salt, as demonstrated for ionic liquids [29]. Furthermore, the conducting salt can also have a plasticizing effect in PIL networks; hence, the viscosities would decrease with the addition of conducting salt [20,21]. This behavior can be observed in Figure 4b for the networks with 2.5 mol% crosslinker content and was also reported by Delhorbe *et al*. [22] for IL homopolymers. When the crosslinker content was increased to 5 mol%, this behavior can no longer be observed. The sample p(AAC6ImC6Cl)/5/0 without conducting salt showed a lower viscosity over the entire temperature range than observed for sample p(AAC6ImC6Cl)/5/5. Due to the higher crosslinking density, it seemed possible that the conducting salt could no longer penetrate the network structure and therefore act only as a disturbing factor, without plasticizing effect. This behavior was also reflected by the *T*_*g*_ values. The sample p(AAC6ImC6Cl)/5/0 displayed a lower *T*_*g*_ than p(AAC6ImC6Cl)/5/5 with conducting salt (by about 5 K).

The temperature dependence of viscosity is commonly fitted by the Vogel-Fulcher-Tamman (VFT) Equation (2) [22,30]

(2) $$\eta =\eta_{0}exp\left[\frac{B}{T-T_{0}}\right]$$

for which *η* is the complex viscosity, T is the absolute temperature, *η*_*0*_ is the viscosity at infinite temperature, B is a constant related to activation energy and *T*_*0*_ is the Vogel temperature, which means the temperature at which the viscosity is infinite, associated with the onset of ion mobility. In equation (2) *η*_*0*_ [Pa*s], *B* [K] and *T*_*0*_ [K] are the fit parameters [22,30–33]. The best fitting parameters and the corresponding correlation coefficient, *R*^*2*^, are listed in Table 2. The spectra of the temperature-depending viscosity of sample p(AAC3ImC4Cl)/5/0 is shown in Figure S5.

**Table 2.** Fitting parameters *η*_*0*_, *B*, *T*_*0*_, and the correlation coefficient R^2^ obtained by the VFT equation (1) for the p(AACXImCYCl)/CL (mol%)/conducting salt (mol%) network samples.

PIL network	*η*_*0*_ [Pa s]	*B* [K]	*T*_*0*_ [K]	*R*^*2*^
p(AAC3ImC4Cl)/2.5/0	1.49	37.97	235.59	0.9935
p(AAC3ImC4Cl)/5/0	1.77	19.50	253.67	0.9859
p(AAC6ImC4Cl)/2.5/0	1.96	8.68	270.50	0.9990
p(AAC6ImC4Cl)/5/0	1.97	7.65	272.30	0.9971
p(AAC6ImC4Cl)/9/0	2.19	2.10	284.12	0.9876
p(AAC6ImC4Cl)/10/0	2.29	1.36	286.13	0.9869
p(AAC6CIm6Cl)/2.5/0	2.38	2.83	277.33	0.9949
p(AAC6ImC6Cl)/2.5/5	2.28	4.88	270.29	0.9962
p(AAC6ImC6Cl)/5/0	2.42	0.83	285.78	0.9942
p(AAC6ImC6Cl)/5/5	2.47	1.16	285.37	0.9928
p(AAC8ImC4Cl)/2.5/0	2.09	5.28	270.16	0.9991

The infinite viscosity *η*_*0*_ increased with both, increasing alkyl spacer length and the crosslinker content. This behavior fitted well with the experimental data. With increasing alkyl-chain length and crosslinker content the viscosities of the samples increased as well. The impact of the alkyl spacer length on the Vogel temperature *T*_*0*_ was more pronounced than the impact of the crosslinker content, indicating that the chemistry of the acrylic substituents played a higher role than the network density, which already could be observed in the literature [34]. In previous years, the relationship between *T*_*g*_ and *T*_*0*_ has been described by Equation (3).

(3) $$T_{0}=T_{g}-50K$$

However, this relationship cannot be applied without doubts since the polymer chain motion must be considered separately from ion mobility [34]. The *T*_*0*_ values of the samples listed here were partially above the *T*_*g*_ values. Numerous ionic liquids, linear homopolymers, gel electrolytes swollen with IL or crosslinked IL containing polyester networks have been studied in the literature [22,31–33,35]. The literature values for the VFT fits differ greatly from the values listed here. However, it is difficult to compare these data because the architectures of the samples are very different. The samples listed here are strongly crosslinked solid-state PIL networks with a limited segmental motion; hence, higher *T*_*0*_ values are to be expected.

In some cases, the Arrhenius Equation (4) is used for fitting the temperature-depending viscosity.

(4) $$\eta =\eta_{\infty }exp\left[\frac{-E_{A}}{R*T}\right]$$

where *η*_*∞*_ is the maximum viscosity, *T* is the absolute temperature, *R* is the universal gas constant and *E*_*A*_ is the activation energy. In the plots of ln*η* versus 1000/T, the curves did not show straight lines; hence, the experimental values did not follow the Arrhenius behavior. As mentioned in the literature, the Arrhenius equation is used for temperature-dependent viscosities below *T*_*g*_, while above *T*_*g*_ the VFT equation is applied [22].

### 3.3. Ionic conductivity of the IL polymer electrolyte networks as a function of temperature

After the glass transition temperatures as well as the viscosity as a function of temperature of the PIL networks have been investigated in detail, the ionic conductivities were also be investigated in the same temperature range (293.15 to 353.15 K) in order to be able to draw conclusions about the influence of structural variations of the networks with respect to their application in battery cells. The requirements for the electrolytes are usually very high. On the one hand, a high degree of flexibility must be achieved, accompanied by high ionic conductivities. On the other hand, the electrolyte must act as a separator with a certain mechanical stability between the electrodes in order to avoid short circuits. In contrast to liquid electrolytes, in which the charge carriers move by free ion diffusion and Brownian motion, the movement of the charge carriers in solid electrolytes is reduced because it takes place *via* ion hopping within the segments [36]. According to the literature, conductivities of 10^−3^ − 10^−4^ S·cm^−1^ are required to provide functioning electrolytes in battery cells. A disadvantage of ionic liquids is their low conductivity at room temperature, which has already been shown in previous investigations [20,21]. The room temperature ionic conductivities of such networks were in the range 10^−6^ S·cm^−1^ or lower. However, it was shown that by constantly increasing the temperature up to 70°C, the ionic conductivities could be increased by two orders of magnitude to 10^−4^ S·cm^−1^. According to Equation (5), by increasing temperature, the viscosity *η* decreases (already shown in section *3.2*.) and with that the mobility *μ* of the networks increases, accompanied by an increase in (ionic) conductivity *σ* (Equation (6)) [37].

(5) $$\mu =\frac{q}{6\pi *\eta *r}$$

(6) σ=n∗q∗μ

where *q* is the charge of the charge carrier, *n* is the density of charge carriers and *r* the radius of the charge carrier.

**Figure 5.**
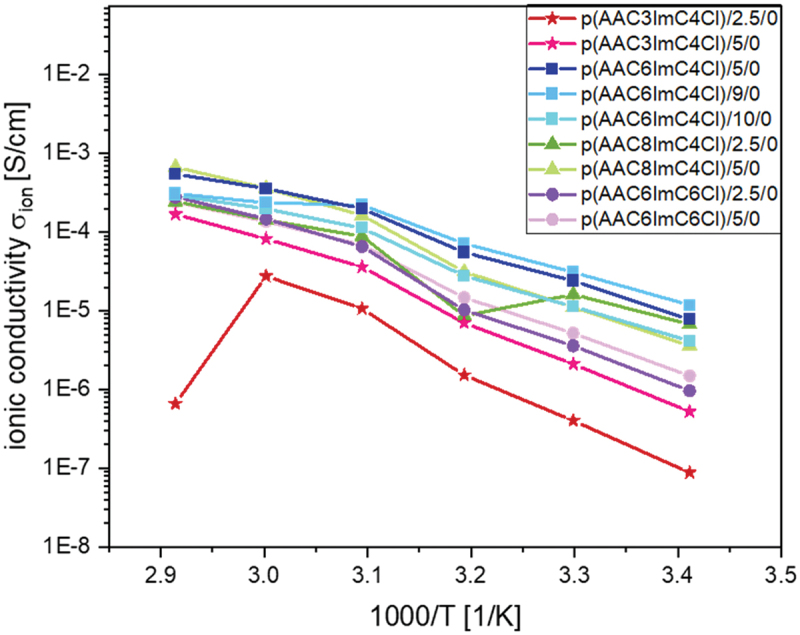
Ionic conductivites of p(AACXImCYCl)/CL (mol%)/conducting salt (mol%) IL networks with different crosslinker content and without conducting salt as a function of temperature.

This behavior will be investigated here for additional samples. Table S1 summarizes the ionic conductivities of all investigated samples. The Nyquist plots of all samples are shown in Figure S6-9. Figure 5 illustrates the measurements of PIL network samples with different amounts of crosslinker and without conducting salt.

In the temperature range from 293.15 to 343.15 K, the ionic conductivities increased strongly with temperature by two orders of magnitude. However, they decreased again (not shown here) at 80°C (353.15 K). Notably, this temperature-dependent behavior is reversible. When cooling down to 20°C, the ionic conductivity of the samples decreased again to the original value. In Figure 5, no direct correlation between the number of alkyl C atoms, *T*_*g*_ and ionic conductivity was observed. Moreover, small changes in the number of crosslinkers showed only a minor influence on the ionic conductivity. Furthermore, it had to be noted that the charge carrier density additionally determined the ionic conductivity. By increasing the crosslinker content, the number of charge carriers (chloride ions) was not significantly altered. The samples p(AAC3ImC4Cl)/2.5/0 and p(AAC8ImC4Cl)/2.5/0 did not show a stable behavior over the entire temperature range. Therefore, a crosslinker content of 5 mol% was used preferentially since these samples showed sufficient stability over the entire temperature range.

**Figure 6.**
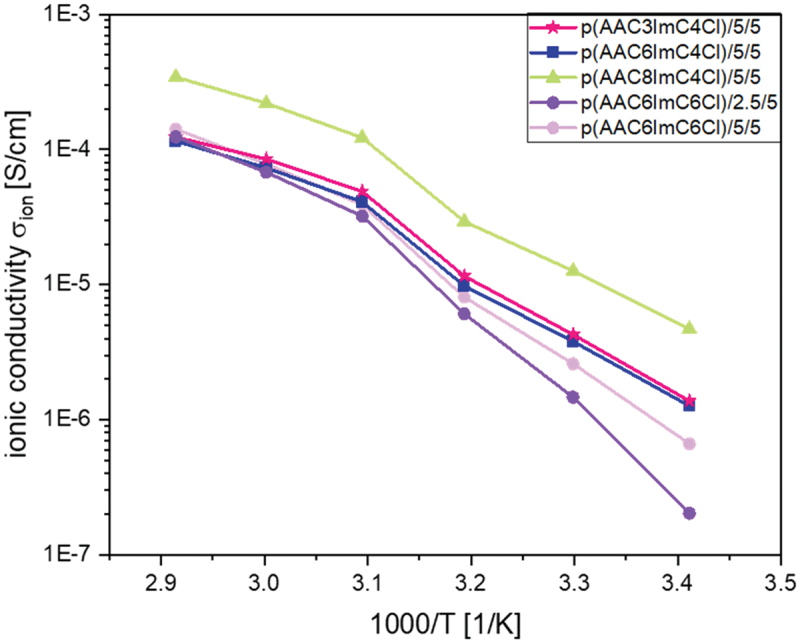
Ionic conductivites of p(AACXImCYCl)/CL (mol%)/conducting salt (mol%) IL networks with 5 mol% conducting salt TBACl as a function of temperature.

When adding 5 mol% TBACl as conducting salt (Figure 6), it was expected that the ionic conductivity would increase. In almost all cases, this behavior could not be observed, except for the samples p(AAC3ImC4Cl)/5/0, p(AAC3ImC4Cl)/5/5, p(AAC8ImC4Cl)/5/0 and p(AAC8ImC4Cl)/5/5. Here, a small increase in ionic conductivity at room temperature could be found when adding 5 mol% conducting salt (compare Figures 5 and 6). This small increase in ionic conductivity disappeared with further increase in temperature. In the literature, this is explained by the fact that the ionic conductivity at low temperature (room temperature) strongly depends on the dynamics of the polymer chains and its impact on the mobility of the free ions, while at higher temperatures, the ionic conductivities rely primarily on the total content of free ions. This was called ‘crossover of *T*_*g*_-controlled to ion concentration-controlled behavior’ [34]. This could also be observed in the viscosity behavior of the samples. It has to be noted that the structure of the polymers studied here is completely different to that studied by Rhoades *et al*. [34] who described this behavior for thiol-ene polymers with different amounts of incorporated IL monomers.

Usually, the temperature dependence of the ionic conductivity is fitted by the Vogel-Fulcher-Tamman (VFT) (Equation (7)):

(7) $$\sigma_{ion}=\sigma_{0}exp\left[\frac{-B}{T-T_{0}}\right]$$

in which *σ*_*ion*_ is the ionic conductivity, *T* is the absolute temperature, *σ*_*0*_ is the infinite temperature conductivity, *B* is a constant related to the activation energy and *T*_*0*_ is the Vogel temperature. In equation (7), *σ*_*0*_ [Pa*s], *B* [K] and *T*_*0*_ [K] are the fit parameters. In the literature, this fitting process is mentioned for a number of imidazolium-containing ionic liquid monomers and their related homopolymers [22,30–33]. For the experimental data obtained for crosslinked ionic liquid networks in this study, the VFT model could not be applied. Therefore, the data were plotted with the Arrhenius model (Equation (8))

(8) $$\sigma =\sigma_{\infty }exp\left[\frac{-E_{A}}{R*T}\right]$$

where *σ*_*∞*_ is the maximum conductivity, *T* is the absolute temperature, *R* is the universal gas constant and *E*_*A*_ is the activation energy. Table 3 summarizes the fitting parameters. The *σ*_*∞*_ values of all samples are in the same range, which could already be shown in Figures 5 and 6. However, the values for the activation energies are different, especially for the samples with 2.5 mol% crosslinker. The *E*_*A*_ values are much higher, which again suggests that the number of crosslinkers is still too low to form stable networks. Thus, with the Arrhenius model, no direct correlation between crosslinker and conducting salt content, alkyl C atom number and *T*_*g*_ values on the ionic conductivity can be found.

**Table 3.** Fitting parameters *σ*_*∞*_, *E*_*A*_, and the correlation coefficient R^2^ obtained using the Arrhenius equation (8) for the p(AACXImCYCl)/CL (mol%)/conducting salt (mol%) network samples (samples written in *italic*: one data point was masked for better R^2^ value).

PIL network	*σ*_*∞*_ [S·cm^−1^]	*E*_*A*_ [J·mol^−1^]	*R*^*2*^
p(AAC3ImC4Cl)/2.5/0	3.50	119.89	0.9931
p(AAC3ImC4Cl)/5/0	3.27	99.43	0.9914
p(AAC3ImC4Cl)/5/5	2.95	79.07	0.9781
p(AAC6ImC4Cl)/5/0	2.92	73.58	0.9857
p(AAC6ImC4Cl)/5/5	2.94	78.90	0.9835
p(AAC6ImC4Cl)/9/0	2.50	56.87	0.9445
p(AAC6ImC4Cl)/10/0	2.92	75.08	0.9818
p(AAC6CIm6Cl)/2.5/0	3.29	99.10	0.9865
p(AAC6ImC6Cl)/2.5/5	3.39	108.91	0.9768
p(AAC6ImC6Cl)/5/0	3.13	88.38	0.9876
p(AAC6ImC6Cl)/5/5	3.18	92.70	0.9867
p(AAC8ImC4Cl)/2.5/0	2.57	60.86	0.9943
p(AAC8ImC4Cl)/5/0	3.22	91.62	0.9887
p(AAC8ImC4Cl)/5/5	2.93	75.41	0.9839

### 3.4. Swelling behavior of selected polymer electrolyte network samples

The swelling behavior of polymer networks is a very important characteristic. Polymer networks cannot be dissolved but may swell in a suitable solvent. Swelling takes place for the polymer segments between network nodes. The (mass-related) degree of swelling *Q*_*m*_ of a network reflects the ratio of the absorbed solvent *m*_*gel*_ compared to the polymeric material *m*_*polymer*_ according to Equation (9) [38]:

(9) $$Q_{m}=\frac{m_{gel}-m_{polymer}}{m_{polymer}}$$

Whether and how much solvent can be incorporated into the network during the swelling process depends on the type of swelling agent, and on the type, shape and size of the materials to be swollen, as well as their interaction that could be described by interaction or solubility parameters [39]. Moreover, the absorption of the solvent is diffusion-controlled. *Q*_*m*_ is a thermodynamic property of the gel and depends on the interaction between polymer segments and solvent, the degree of crosslinking, salt concentration, pressure, temperature and pH value [40,41]. With these in mind, the samples were analyzed for their swelling behavior as a function of the crosslinker and conducting salt content. In a suitable solvent, the following dependence on the crosslinking density *ν*_*e*_ applies for the mass-related degree of swelling *Q*_*m*_ (Equation (10)) [38]:

(10) $$\nu_{e}∼\frac{1}{Q_{m}}$$

**Figure 7.**
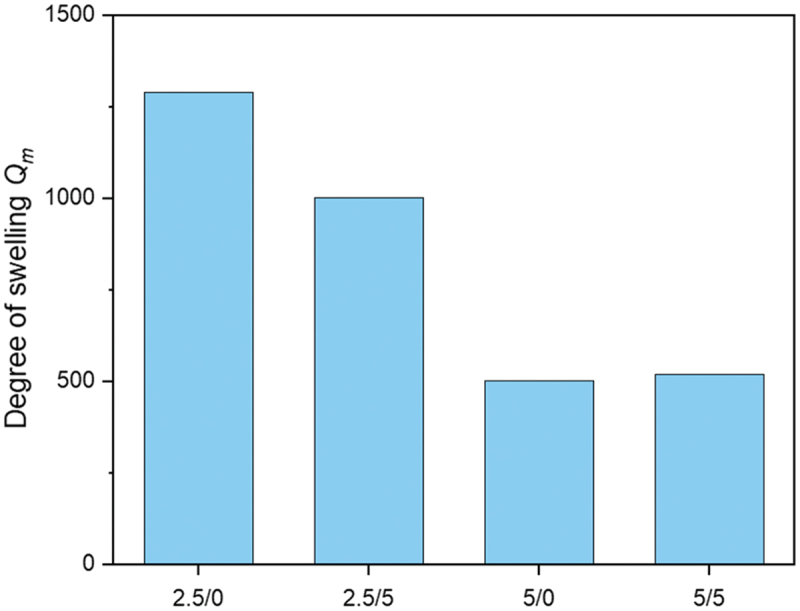
Swelling behavior of p(AAC6ImC6Cl)/CL (mol%)/conducting salt (mol%) IL networks in water (analyzed after one week of swelling).

The samples were swollen for 1 week in the medium water. Normally, the samples are swollen in the measurements until a mass equilibrium is reached. Figure 7 shows the swelling behavior of p(AAC6ImC6Cl) network samples with different amounts of crosslinker and conducting salt. As can be seen, water is a suitable swelling agent because it has similar polarities as the ionic monomer units and thus causes strong interactions between solvent and polymer segments. Degrees of swelling *Q*_*e*_ until values of about 1250 were reached, indicating the strong interaction between ionic network segments and the polar solvent water. As expected, *Q*_*m*_ decreased with increasing crosslinker concentration. The effect of the conducting salt concentration is higher in the samples with 2.5 mol% crosslinker due to the lower crosslinking density. In p(AAC6ImC6Cl)/5/5 the conducting salt has only a minor influence on the swelling behavior. An equilibrium was not achieved in the samples examined after 1 week. However, this fact is not important with regard to the application as polymer electrolytes in batteries. The swelling of the polymer is used to widen the meshes for the charge carriers to be moved so that they can diffuse better through the electrolyte. This should result in a strong increase in ionic conductivity.

The ionic conductivity of the three gels swollen in water was determined at 20°C. The Nyquist plots of the three samples are shown in Figure S10. As summarized in Table 4, swelling had a significant effect on the ionic conductivity and values of 10^−2^ S·cm^−1^ can be achieved. These values clearly exceeded the values at 70°C. It can be noted that sufficiently high ionic conductivity values could already be achieved at 20°C by means of swelling in water, which is an excellent prerequisite for battery application.

**Table 4.** Ionic conductivities (at 20°C) of p(AAC6ImC6Cl)/CL (mol%)/conducting salt (mol%) IL network samples before and after swelling.

PIL network	*σ*_*ion, before*_ [S·cm^−1^]	*σ*_*ion, after*_ [S·cm^−1^]
p(AAC6CIm6Cl)/2.5/0	9.67·10^−7^	7.71·10^−2^
p(AAC6ImC6Cl)/2.5/5	2.03·10^−7^	2.30·10^−2^
p(AAC6ImC6Cl)/5/5	6.64·10^−7^	5.10·10^−2^

For the following battery tests, the sample p(AAC6ImC4Cl)/5/5 was swollen in the ionic liquid 1-hexyl-3-methylimidazolium chloride for 1 week (Figure S11). The swelling in the IL was not as strong as in water and reached 145%. Nevertheless, it can be assumed that a certain interaction with the polymer matrix takes place. Therefore, the IL was employed in the following battery test.

### 3.5. Battery test with the polymer electrolyte networks p(AAC6ImC4Cl)/5/5 and ionic liquid 1-hexyl-3-methylimidazolium chloride

**Figure 8.**
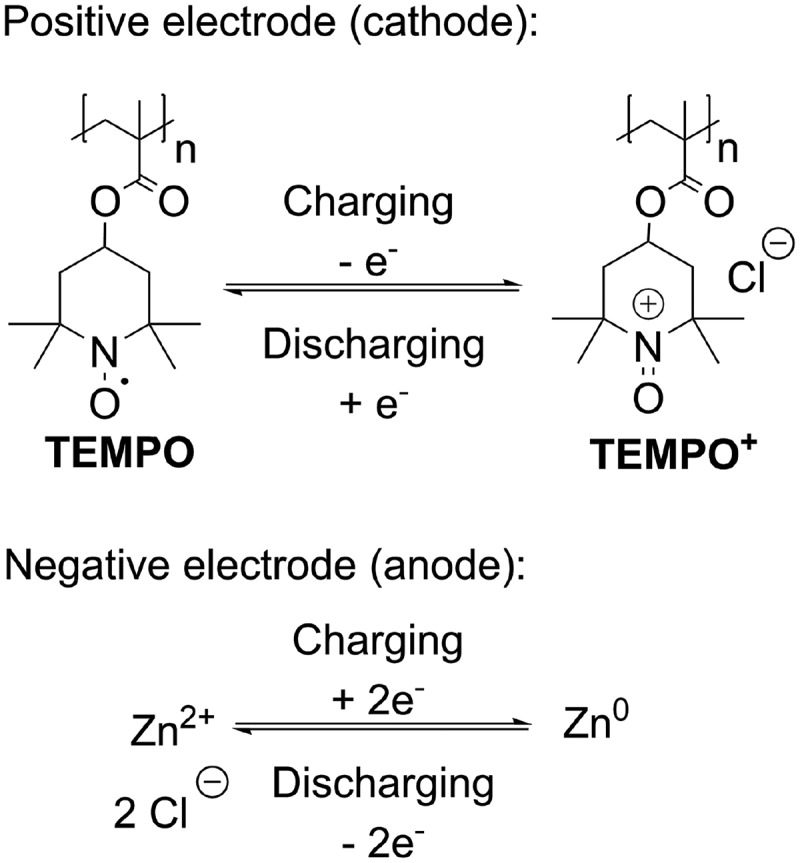
Redox reactions of p(TEMPO-MA)^+^/p(TEMPO-MA) and Zn^2+^/Zn^0^ redox couple.

The redox couple p(TEMPO-MA)^+^/p(TEMPO-MA) (PTMA) and Zn^2+^/Zn^0^ is well known in the literature for semi-organic hybrid flow batteries (aqueous/non-aqueous) [17,42–44] and was used here. Considering this, the zinc-containing conducting salt Zn(ClO_4_)_2_·6 H_2_O was employed to increase the concentration of Zn^2+^ ions in the electrolyte. Figure 8 shows the redox reactions occurring at the electrodes p(TEMPO-methacrylate) and zinc.

In a first test experiment, the electrodes p(TEMPO-MA) and zinc and a liquid electrolyte (50 µl of a solution of 1 M Zn(ClO_4_)_2_·6 H_2_O in a mixture of EC/DMC in a 1/1 mol/mol ratio with 74 mg of linear homopolymer p(AAC6ImC4Cl)) and a standard Celgard separator were tested in a Swagelok cell from 0.5 to 2 V at 1C for 100 cycles. Even after different preparation variations, this cell was not working. At the beginning, the charge factor was over 100% and there was no typical discharge behavior (Figure S12).

Based on the results, the monomer AAC6ImC4Cl with 5 mol% crosslinker and 5 mol% conducting salt was selected for initial cycling tests with an electrochemical stability over a wide voltage range (−3 to 3 V) [20,21]. According to section 3.4, the PIL networks showed a good swelling behavior in water, so a second test experiment was performed using the electrolyte p(AAC6ImC4Cl) with 5 mol% CL, 5 mol% conducting salt and 20 wt% water (based on the monomer content). The polymer acted both as an electrolyte and a separator. The cell was charged/discharged in a voltage range from 0.5 to 1.5 V at 0.1C for 500 cycles. The charge factor of about 60% and capacity values with 10^−4^ mAh were still low, but according to the voltage profile it was a step forward compared to the first test (Figure S13).

**Figure 9.**
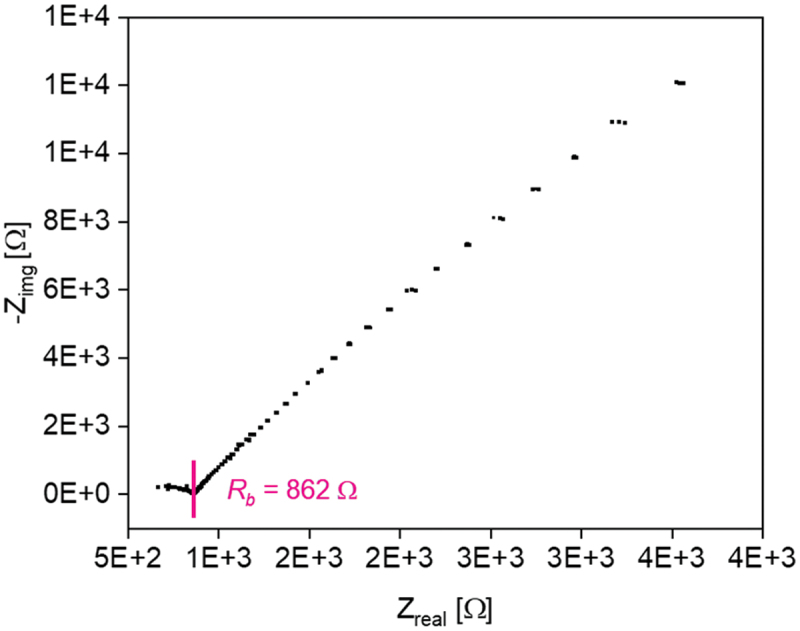
Nyquist plot of the PEL with crosslinker, conducting salt and ionic liquid.

In the literature, ionic liquids were used to improve the performance of a battery [17–19]. Hence, the chloride-containing ionic liquid 1-hexyl-3-methylimidazolium chloride was added in the ratio vol_IL_/vol_monomer_ 2/1 to provide higher battery capacity values, higher ionic conductivity and to increase the concentration of counter ions for the redox processes at the electrodes. With that, an ionic conductivity of 1.67·10^−3^ S·cm^−1^ was achieved. The addition of the IL was a key factor to increase the performance of the battery cell. The setup of this battery is illustrated in Figure S14. Figure 9 shows the Nyquist plot obtained with the studied ionic liquid-based polymer electrolyte (PEL) p(AAC6ImC4Cl)/5/5 with 5 mol% CL, 5 mol% Zn(ClO_4_)_2_·6 H_2_O and ionic liquid before the cycling test.

**Figure 10.**
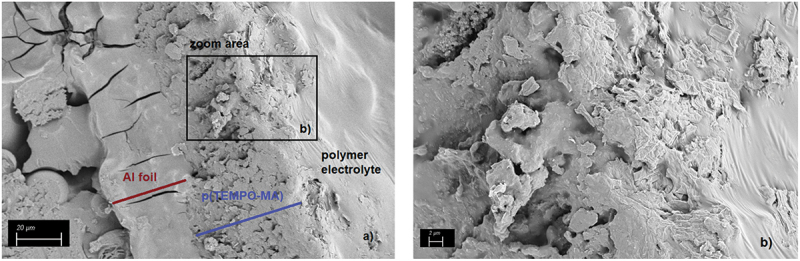
SEM images taken at a) the cross-section of the cathode p(TEMPO-MA) penetrated with PEL, b) zoom-in.

Scanning electron microscopy was used to test whether the electrolyte completely penetrated the active material of the p(TEMPO-MA) electrode. Figure 10 shows the SEM images of a cross-section of the p(TEMPO-MA) electrode penetrated with the PEL after one-hour diffusion time. It is clearly visible that the electrolyte penetrated completely into the active material during this time, since the porous structures are covered with the electrolyte layer up to the aluminum foil. This is considered an important prerequisite for the redox processes taking place in the cell so that it can be assumed that the charge carriers can migrate from one electrode to the other through the PEL.

**Figure 11.**
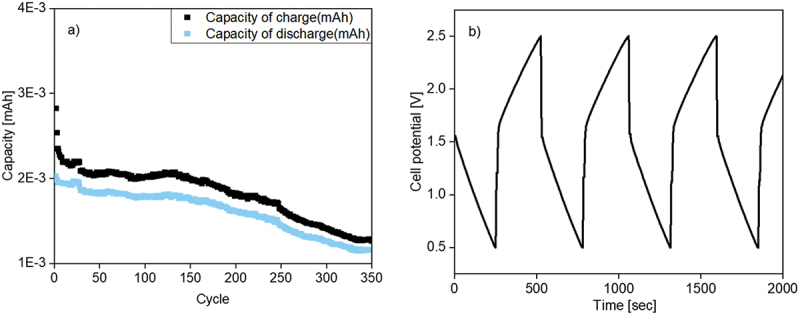
p(TEMPO-MA)/zinc battery with polymer electrolyte, a) charge/discharge capacities during cycling, b) selected charge/discharge curves.

In the galvanostatic cycling test, the electrodes had the same diameter of 16.4 mm and the cell was charged/discharged between 0.5 and 2.5 V at a C-rate of 0.1C for 350 cycles in total. The charge factor was about 90% over the entire test (Figure S15). Despite IL was added, the charge/discharge capacities were lower than those obtained earlier with other systems by Muench *et al.* [19] (Figure 11a). The charge/discharge curves of cycle 335–337 are shown in Figure 11b. The vertical curve at the beginning of the half cycle suggested redox reactions of the active materials. The capacitances were one order of magnitude higher (10^−3^ mAh) compared to the second test experiment. The resistances in the cells had a significant influence on the overall performance of the battery at low C rates.

To increase the capacitances in the cell and to reduce the resistances of the electrode/electrolyte interfaces, the PEL could be swollen in water (which showed a significant effect on the ionic conductivity of the electrolyte shown in Section 3.4) before incorporating them in the cell. Furthermore, the cathode p(TEMPO-methacrylate) could be substituted by the more polar p(TEMPO-amide). The incorporation of the IL also showed an increase in ionic conductivity. Since the conducting salt introduced additional perchlorate anions into the system, it would be conceivable to use the respective IL with perchlorate counter ions instead of chloride ions. Chloride ions are less dissociated and less mobile [45], which was assumed to have an influence on the ionic conductivity as well as the charging and discharging processes. However, these possibilities remain to be studied in the future and are beyond the scope of this contribution, as we focus mainly on structure–property relationships. Battery optimization tests are already in progress and the results so far are promising. However, it can be stated at this point that the tests shown proved the suitability of the polymer electrolytes studied in principle.

## 4. Conclusion

Polymer electrolyte networks containing basic units of ionic liquid monomers with long alkyl spacers (*i.e*., acrylic monomers with alkyl substituents coupled to imidazolium groups) with stepwise varied chemical structure were prepared and analyzed with different methods to elucidate structure–property relationships with respect to the influence of chemical structure on the glass transition temperatures, the rheological behavior and the ionic conductivity, as well as to find out the suitability of the networks to act as electrolytes in polymer organic redox batteries.

Radical UV polymerization of IL monomers with structural variations in combination with a fixed crosslinker at different concentrations led to polymer networks with tailored properties. A clear influence of both monomer structure and crosslinker content on the glass transition temperatures of the resulting networks was observed. The *T*_*g*_ values of all investigated networks were far below 0°C, a suitable prerequisite to achieve a high mobility of the charge carriers (chloride ions) at room temperature. The *T*_*g*_s decreased with increasing alkyl spacer length and increased with increasing crosslinker content. Samples with comparable crosslinker content showed a clear tendency that increasing number of methylene groups in the IL monomer units reduced the glass transition temperatures. The conducting salt also had a significant effect on the *T*_*g*_s, as the addition of 5 mol% TBACl resulted in almost all samples in a drop of the glass transition temperature of around 10–20 K.

The C atom number, crosslinker and conducting salt content also had a significant impact on the rheological behavior. For networks p(AAC6ImC4Cl)/CL (mol%)/conducting salt (mol%) with different network density (different crosslinker content), the viscosity was almost comparable at lower temperatures and started deviating at about 50°C. The higher the network density, the higher the viscosity and mechanical strength of the materials at higher temperatures. The total number of C atoms (in alkyl spacer X plus *N*-alkyl substituent Y) played a decisive role for the viscosity. As the number of alkyl groups increased, the viscosity of the networks at 80°C also increased. This behavior was not observed at room temperature.

The main focus of this study was directed to the influence of chemical structure and temperature on the ionic conductivities. At room temperature, the ionic conductivities of the polymer electrolytes were in the range of 10^−6^ to 10^−8^ S·cm^−1^. No significant influence of C-atom number, crosslinker and conducting salt content could be observed at this temperature. At 70°C, the ionic conductivities of all investigated samples increased by two orders of magnitude and partly reached values of 10^−4^ S·cm^−1^, values that could already be achieved in our previous work [20,21]. Notably, it was possible to further increase the conductivity values of selected samples to 10^−2^ S·cm^−1^ (five orders of magnitude higher than the values measured at 20°C) by swelling the network films in water for 1 week. Hence, the ionic conductivity showed a significant influence of the water content in the network.

According to the results, networks with the monomer AAC6ImC4Cl and with 5 mol% crosslinker BAAP UV-polymerized with TPO as photoinitiator proved to be the optimal choice for the desired polymer electrolyte.

The galvanostatic charge/discharge experiments of a p(TEMPO-MA)/zinc battery with addition of ionic liquid in the polymer electrolyte system proved that a working battery cell could be achieved. The addition of an IL seemed to be helpful for the overall performance of the cell. However, it has to be stated that the charge/discharge capacity of the cell needs to be further improved. Different methods to achieve this were recognized.

With this together with the results of the previous work, we have been able to elaborate in great detail the structure–property relationships of the different networks with respect to battery applications so that the basic knowledge for efficient transfer to the battery system is now available. In the next steps, the active materials, the interphases in the battery and the interaction of all components have to be optimized to come closer to the theoretical capacity values. Experiments on this are already in progress but would go beyond the scope of this work.

## Supplementary Material

Supplemental Material

## Data Availability

The data that support the findings of this study are available in the supplementary material of this article (Supporting Information).
