# Supplementary material for: Influencing ionic conductivity and mechanical properties of ionic liquid polymer electrolytes by designing the chemical monomer structure
Source: Des Monomers Polym. 2023 Oct 11;26(1):198–213. doi: 10.1080/15685551.2023.2267235 (PMC10569356; doi:10.1080/15685551.2023.2267235)
Supplement: Supplemental Material [file TDMP_A_2267235_SM1239.docx]

Supporting Information

Influencing ionic conductivity and mechanical properties of ionic liquid polymer electrolytes by designing the chemical monomer structure

*Lisa Ehrlich^a,b^, Doris Pospiech^a^*, Petra Uhlmann^a^, Felix Tzschöckell^c^, Martin D. Hager^c^, Brigitte Voit^a,b^*

1. **DSC curves**
2. **Rheology: complex viscosity curves**
3. **EIS data and spectra of temperature-depending measurements**
4. **EIS spectra after swelling**
5. **Battery tests**
6. **DSC curves**

**
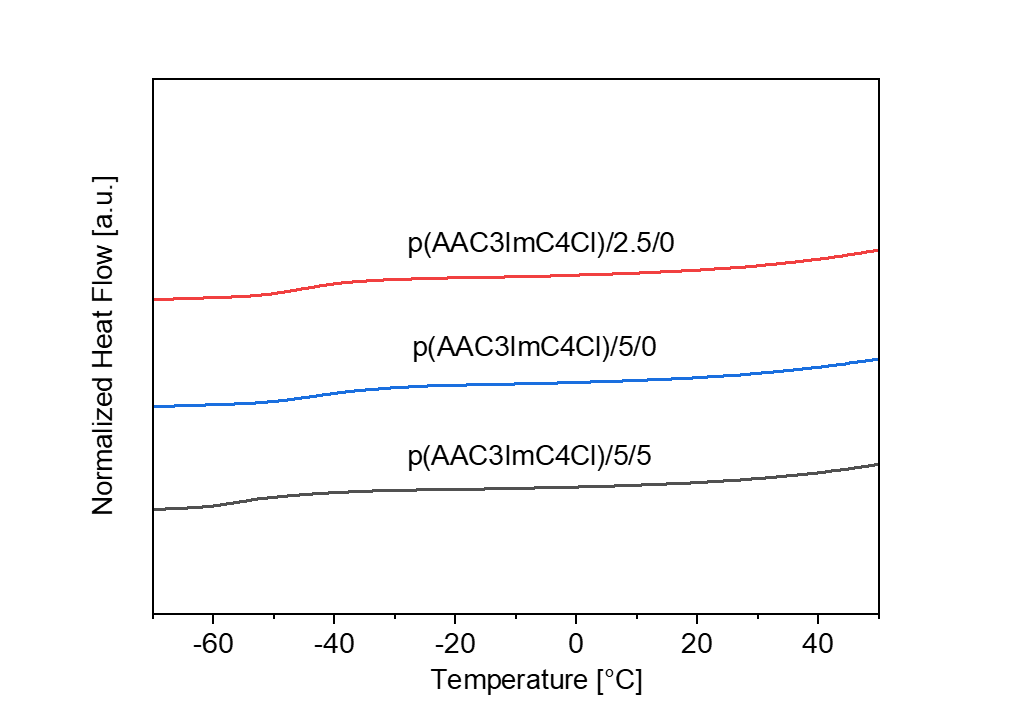
**

**Figure S1.** DSC curves of the p(AAC3ImC4Cl)/ CL (mol%)/ conducting salt (mol%) IL networks.


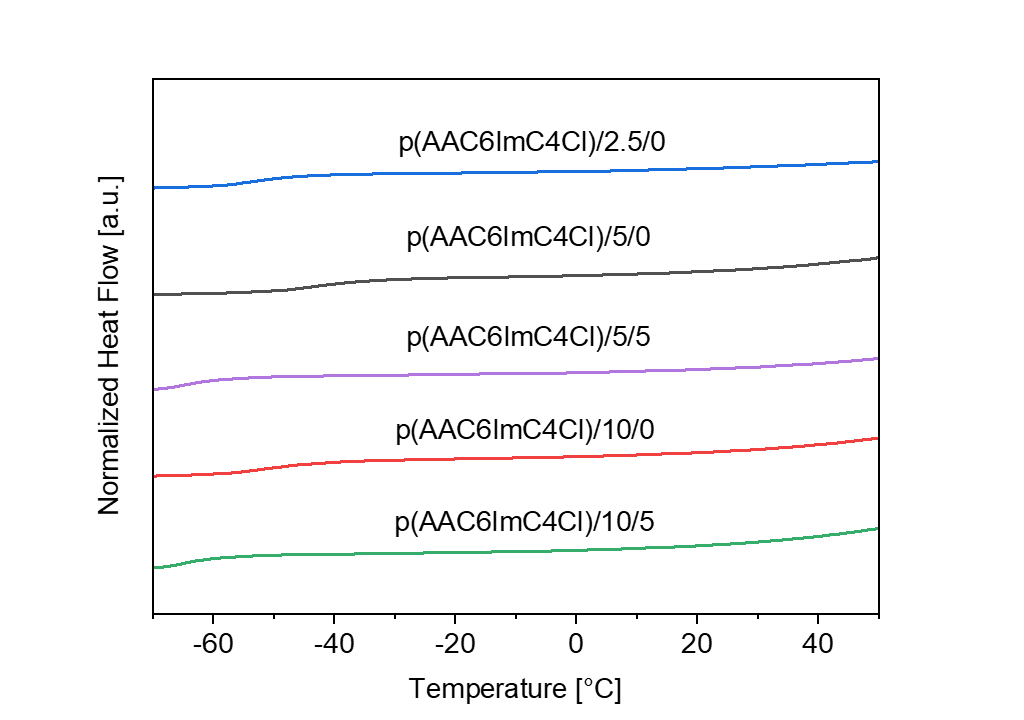


**Figure S2.** DSC curves of the p(AAC6ImC4Cl)/ CL (mol%)/ conducting salt (mol%) IL networks without and with conducting salt.

**
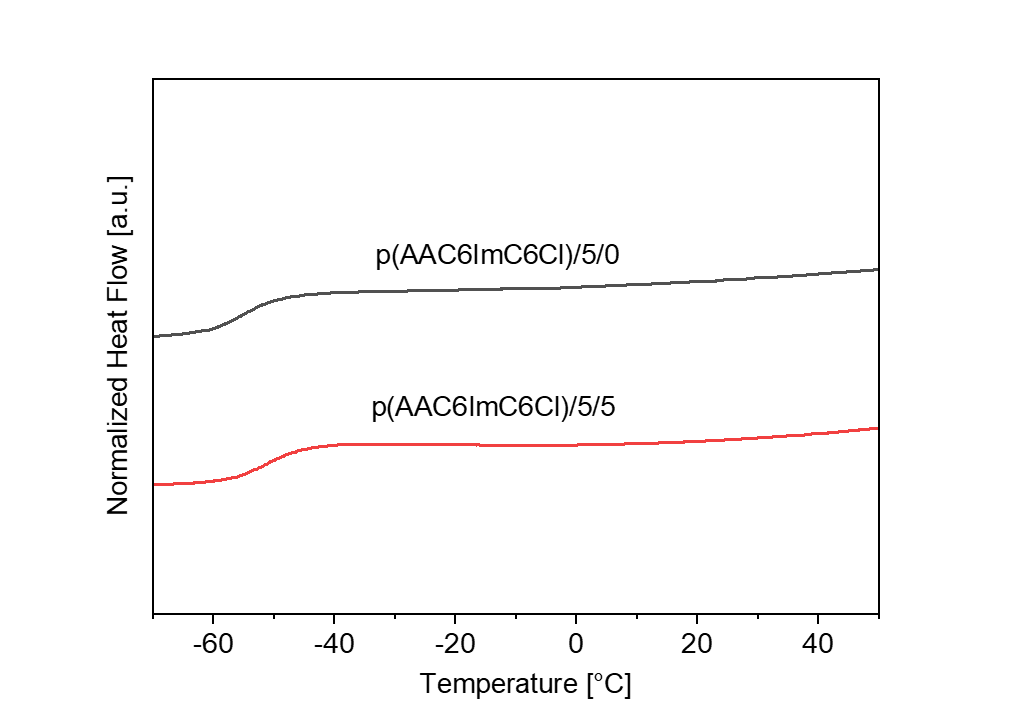
**

**Figure S3.** DSC curves of the p(AAC6ImC6Cl)/ CL (mol%)/ conducting salt (mol%) IL networks without and with conducting salt.

**
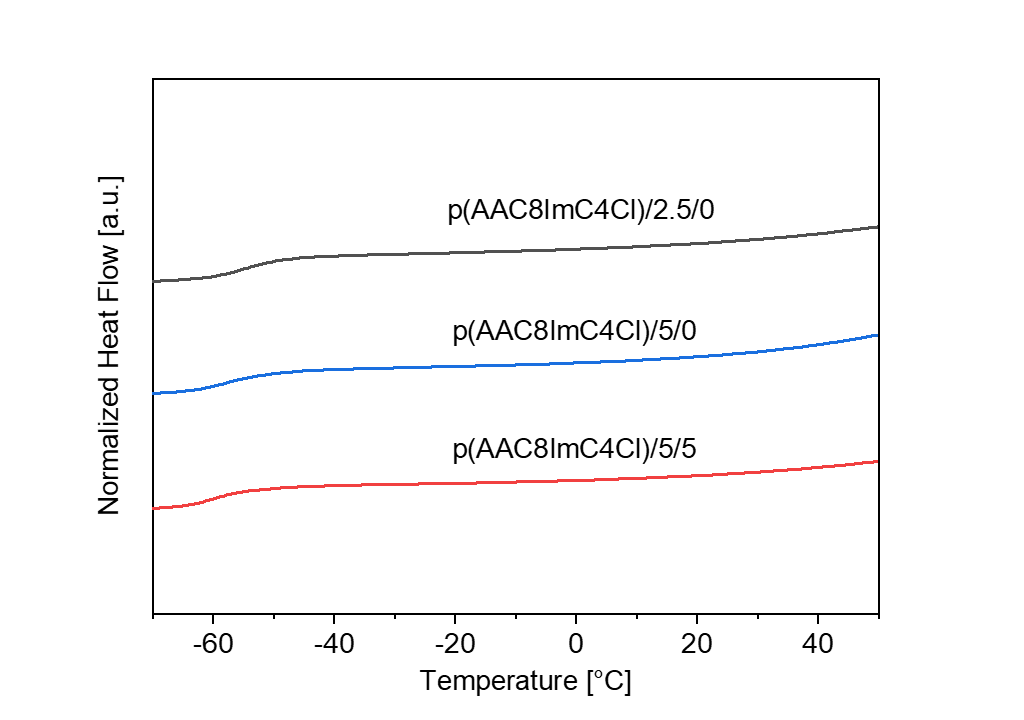
**

**Figure S4.** DSC curves of the p(AAC8ImC4Cl)/ CL (mol%)/ conducting salt (mol%) IL networks without and with conducting salt.

1. **Rheology: complex viscosity curves**

**
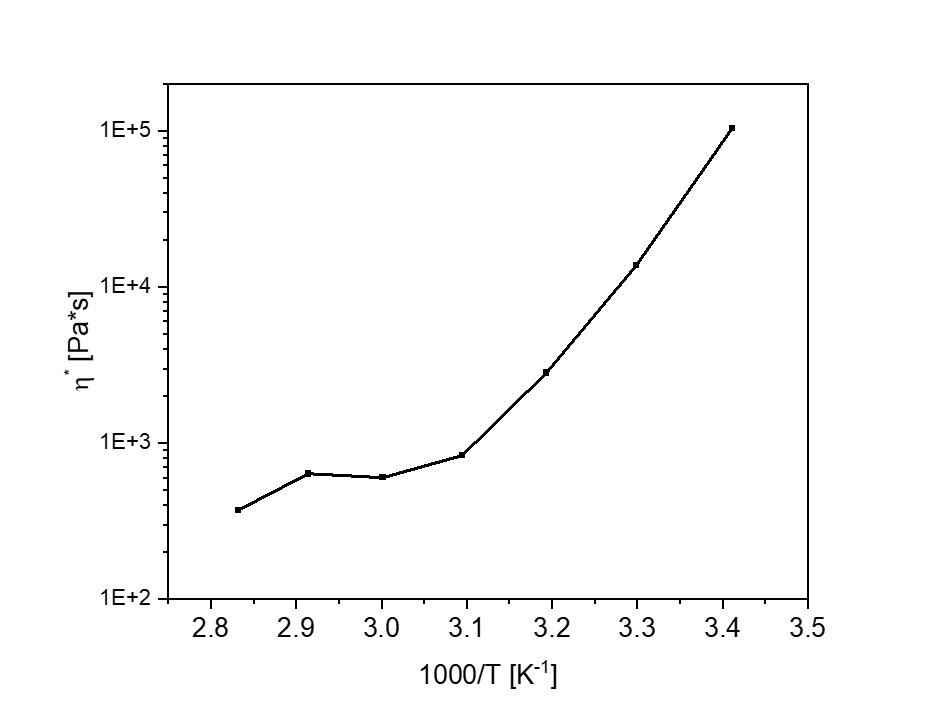
**

**Figure S5.** Complex viscosity as a function of temperature of the p(AAC3ImC4Cl)/5/0 IL network.

1. **EIS data and spectra of temperature depending measurements**

**Table S1**. Ionic conductivities in dependence of temperature of all investigated PIL networks p(AACXImCYCl)/ CL (mol%)/ conducting salt (mol%) (samples written in *italic:* already published in Ehrlich *et al.*[20]*).*

| **PIL network** | ***σ_ion_* [S·cm^-1^] at temperature [°C]** | | | | | |
| --- | --- | --- | --- | --- | --- | --- |
|  | ***20*** | ***30*** | ***40*** | ***50*** | ***60*** | ***70*** |
| p(AAC3ImC4Cl)/2.5/0 | 8.77·10^-8^ | 4.03·10^-7^ | 1.52·10^-6^ | 1.07·10^-5^ | 2.75·10^-5^ | 6.60·10^-7^ |
| p(AAC3ImC4Cl)/5/0 | 5.26·10^-7^ | 2.10·10^-6^ | 7.04·10^-6^ | 3.60·10^-5^ | 8.19·10^-5^ | 1.68·10^-4^ |
| *p(AAC3ImC4Cl)/5/5* | 1.38·10^-6^ | 4.27·10^-6^ | 1.16·10^-5^ | 4.90·10^-5^ | 8.49·10^-5^ | 1.24·10^-4^ |
| *p(AAC6ImC4Cl)/5/0* | 7.78·10^-6^ | 2.41·10^-5^ | 5.52·10^-5^ | 1.98·10^-4^ | 3.58·10^-4^ | 5.49·10^-4^ |
| *p(AAC6ImC4Cl)/5/5* | 1.27·10^-6^ | 3.80·10^-6^ | 9.78·10^-6^ | 4.10·10^-5^ | 7.30·10^-5^ | 1.16·10^-4^ |
| p(AAC6ImC4Cl)/9/0 | 1.18·10^-5^ | 3.10·10^-5^ | 7.16·10^-5^ | 2.20·10^-4^ | 2.36·10^-4^ | 3.09·10^-4^ |
| p(AAC6ImC4Cl)/10/0 | 2.27·10^-5^ | 6.15·10^-5^ | 1.50·10^-4^ | 4.92·10^-4^ | 8.49·10^-4^ | 1.51·10^-3^ |
| p(AAC6CIm6Cl)/2.5/0 | 9.67·10^-7^ | 3.59·10^-6^ | 1.03·10^-5^ | 6.54·10^-5^ | 1.47·10^-4^ | 2.80·10^-4^ |
| p(AAC6ImC6Cl)/2.5/5 | 2.03·10^-7^ | 1.47·10^-6^ | 6.09·10^-6^ | 3.21·10^-5^ | 6.79·10^-5^ | 1.24·10^-4^ |
| p(AAC6ImC6Cl)/5/0 | 1.49·10^-6^ | 5.18·10^-6^ | 1.47·10^-5^ | 6.85·10^-5^ | 1.37·10^-4^ | 2.42·10^-4^ |
| p(AAC6ImC6Cl)/5/5 | 6.64·10^-7^ | 2.60·10^-6^ | 8.08·10^-6^ | 3.89·10^-5^ | 7.82·10^-5^ | 1.42·10^-4^ |
| p(AAC8ImC4Cl)/2.5/0 | 6.77·10^-6^ | 1.62·10^-5^ | 8.62·10^-6^ | 8.83·10^-5^ | 1.43·10^-4^ | 2.44·10^-4^ |
| p(AAC8ImC4Cl)/5/0 | 3.60·10^-6^ | 1.11·10^-5^ | 3.14·10^-5^ | 1.63·10^-4^ | 3.62·10^-4^ | 6.71·10^-4^ |
| *p(AAC8ImC4Cl)/5/5* | 4.71·10^-6^ | 1.26·10^-5^ | 2.93·10^-5^ | 1.22·10^-4^ | 2.20·10^-4^ | 3.43·10^-4^ |

| 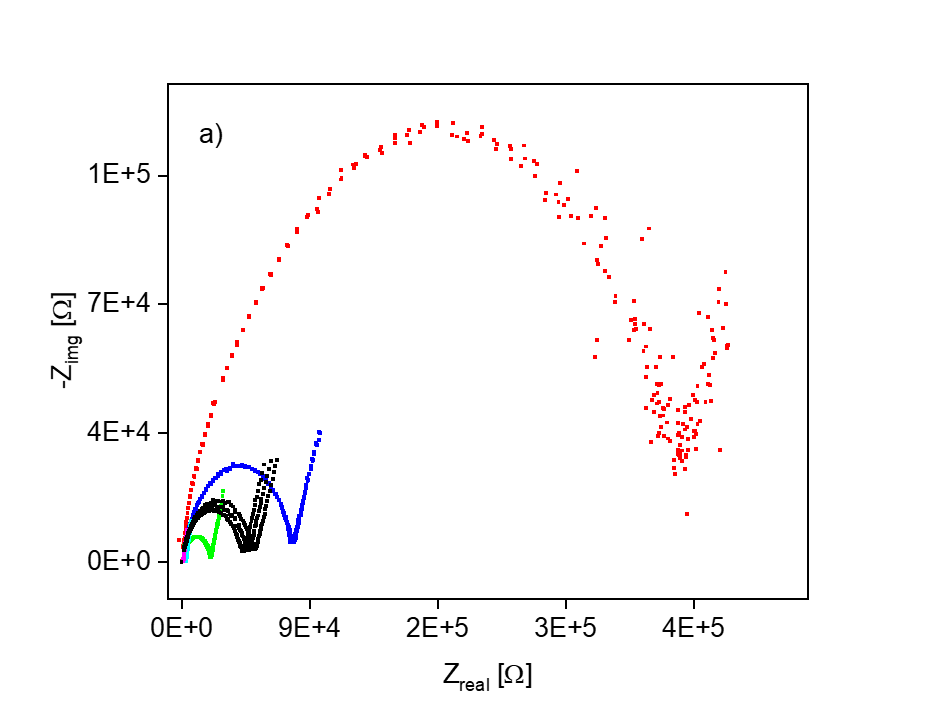 | **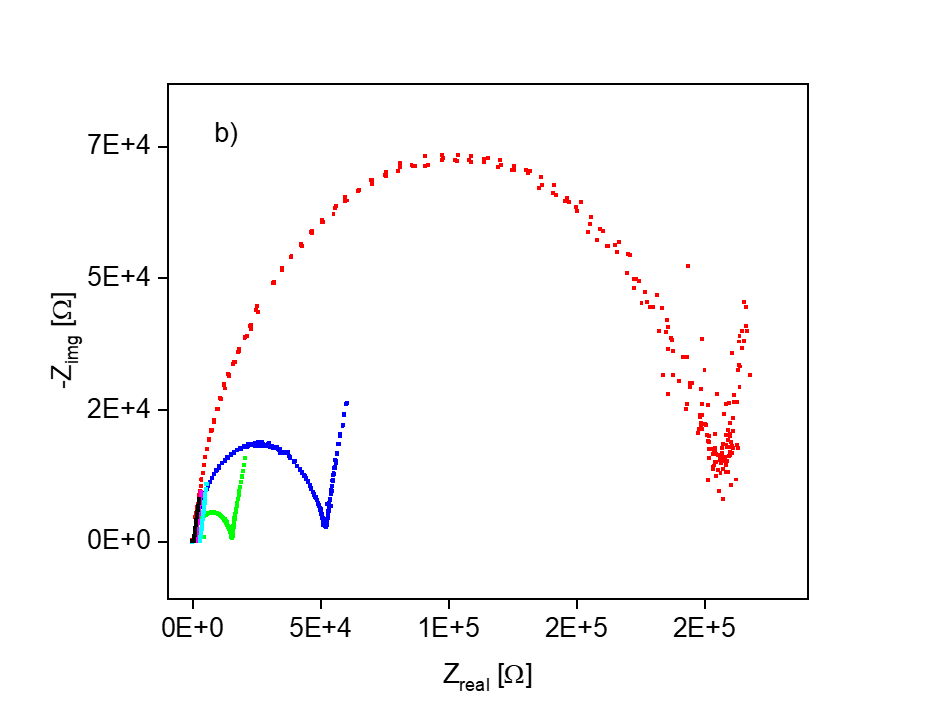** |
| --- | --- |
| **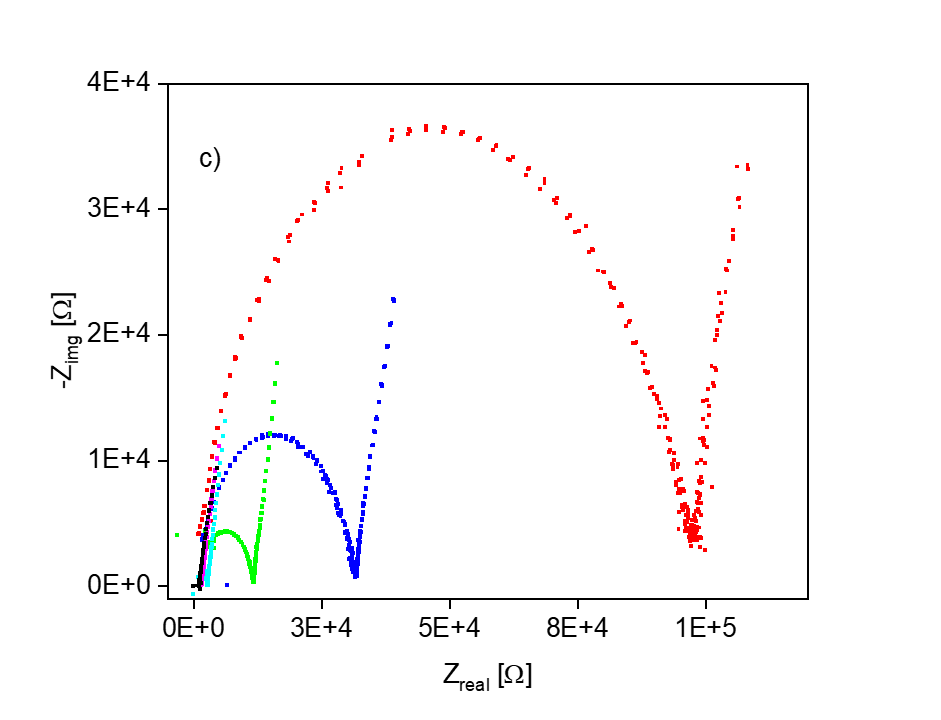** | |

**Figure S6.** Nyquist plots of the temperature-depending EIS measurements of p(AAC3ImC4Cl)/ CL (mol%)/ conducting salt (mol%) IL networks: a) p(AAC3ImC4Cl)/2.5/0, b) p(AAC3ImC4Cl)/2.5/5, c) p(AAC3ImC4Cl)/5/5 (red: 20 °C, blue: 30 °C, green: 40 °C, cyan: 50 °C, magenta: 60 °C, black: 70 °C).

| **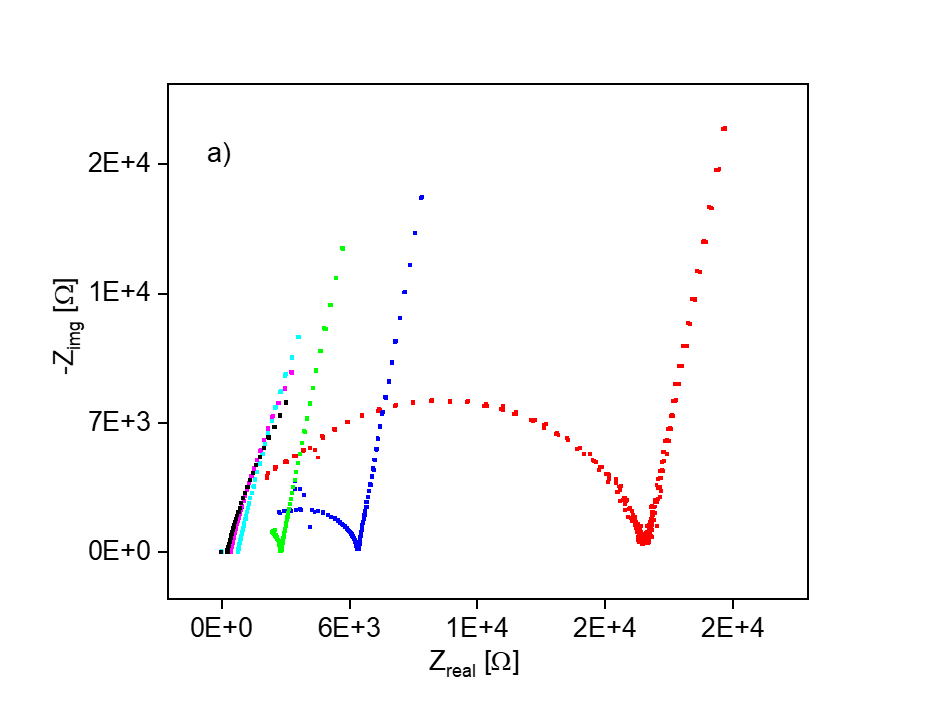** | **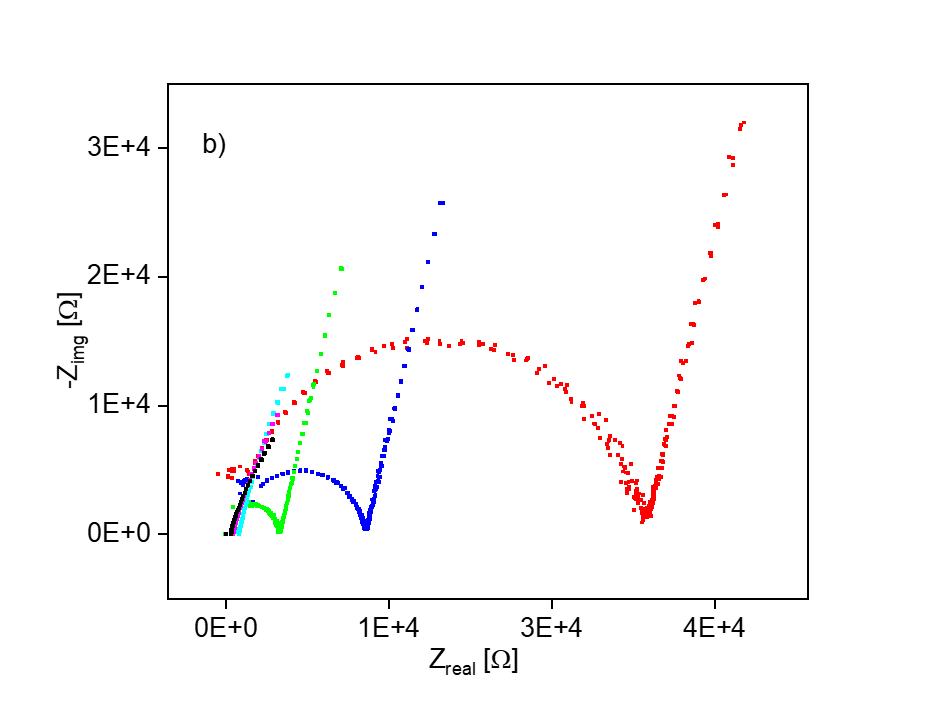** |
| --- | --- |
| **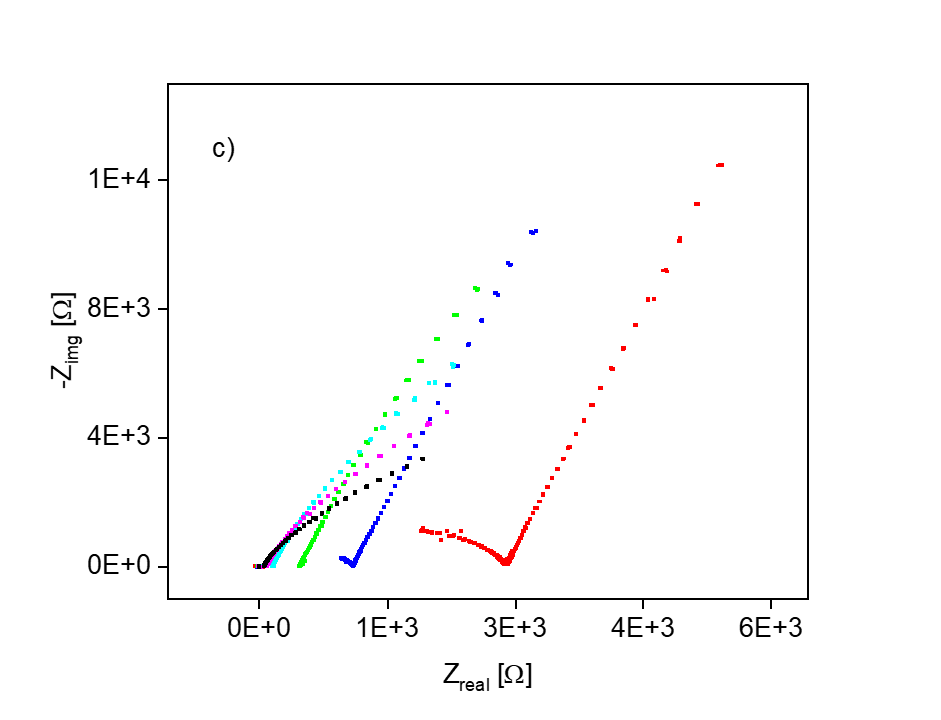** | **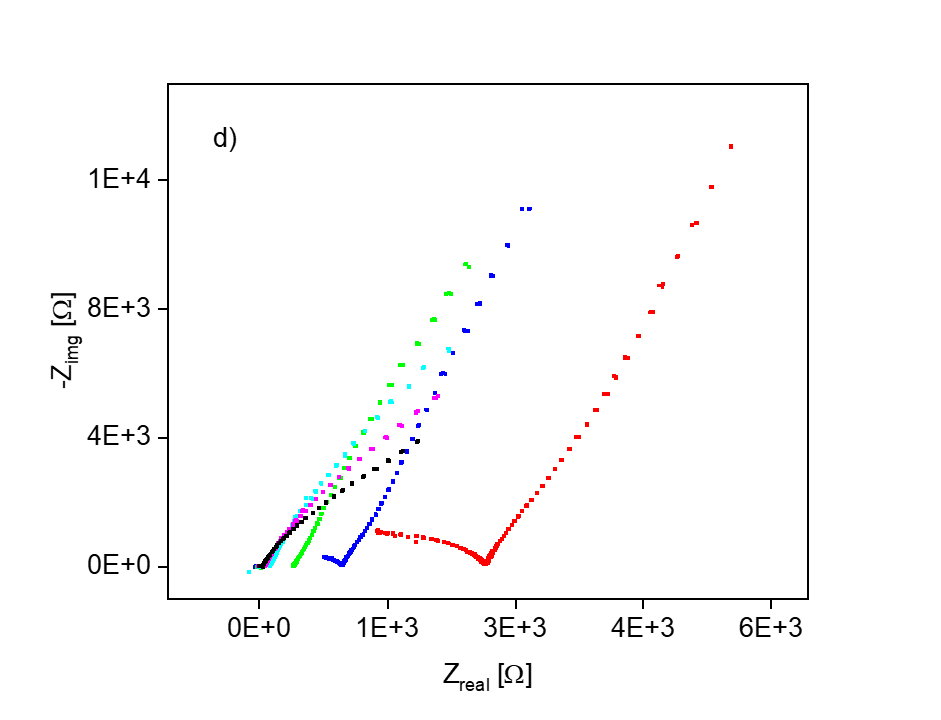** |

**Figure S7.** Nyquist plots of the temperature-depending EIS measurements of p(AAC6ImC4Cl)/ CL (mol%)/ conducting salt (mol%) IL networks: a) p(AAC6ImC4Cl)/5/0, b) p(AAC6ImC4Cl)/5/5, c) p(AAC6ImC4Cl)/9/0, d) p(AAC6ImC4Cl)/10/0 (red: 20 °C, blue: 30 °C, green: 40 °C, cyan: 50 °C, magenta: 60 °C, black: 70 °C).

| 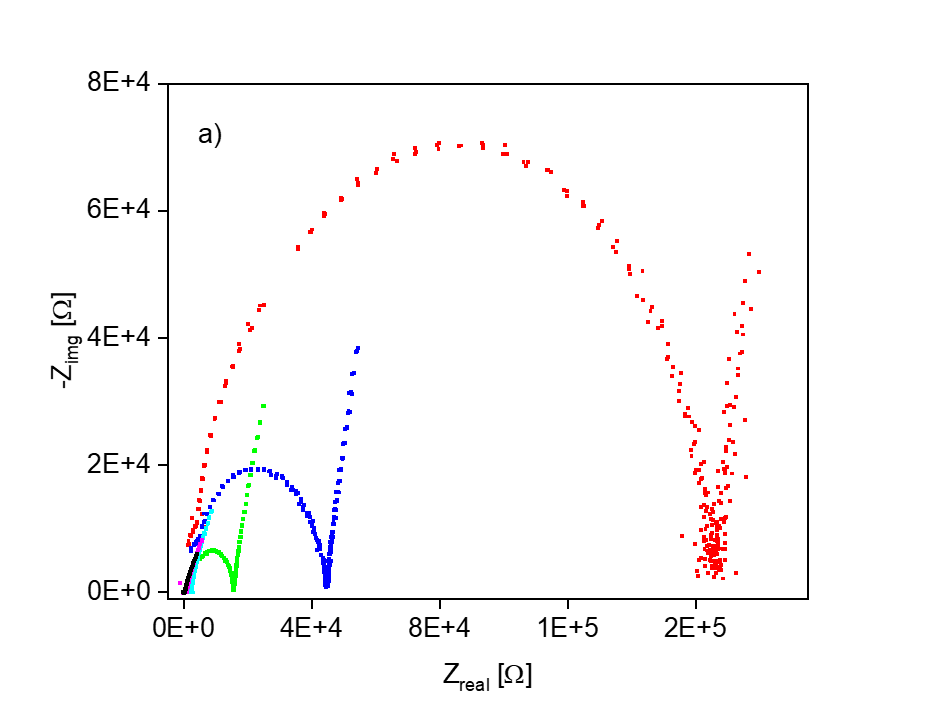 | **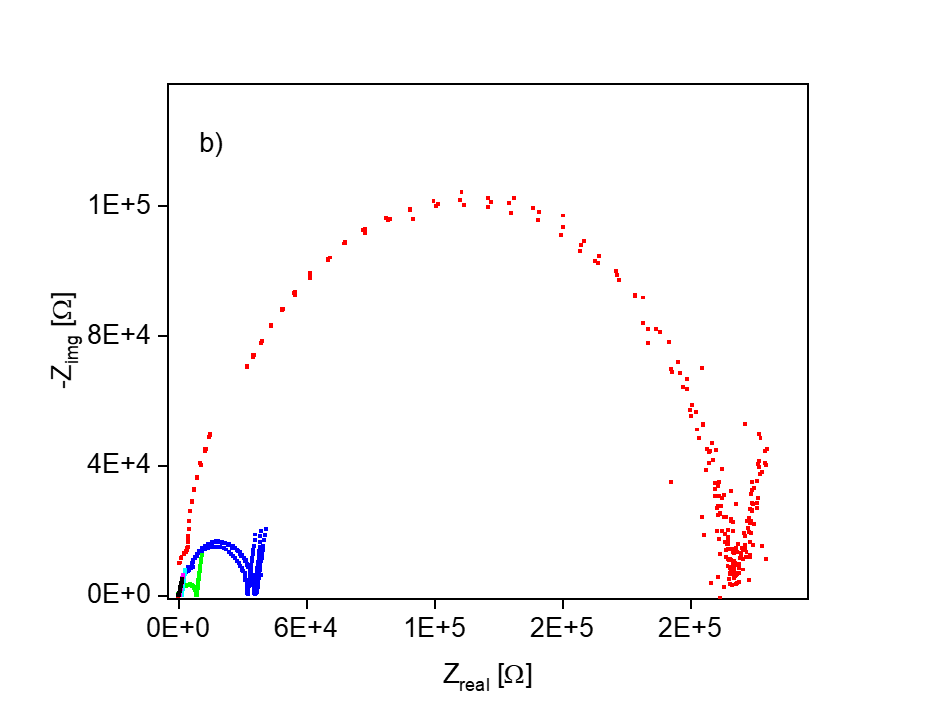** |
| --- | --- |
| **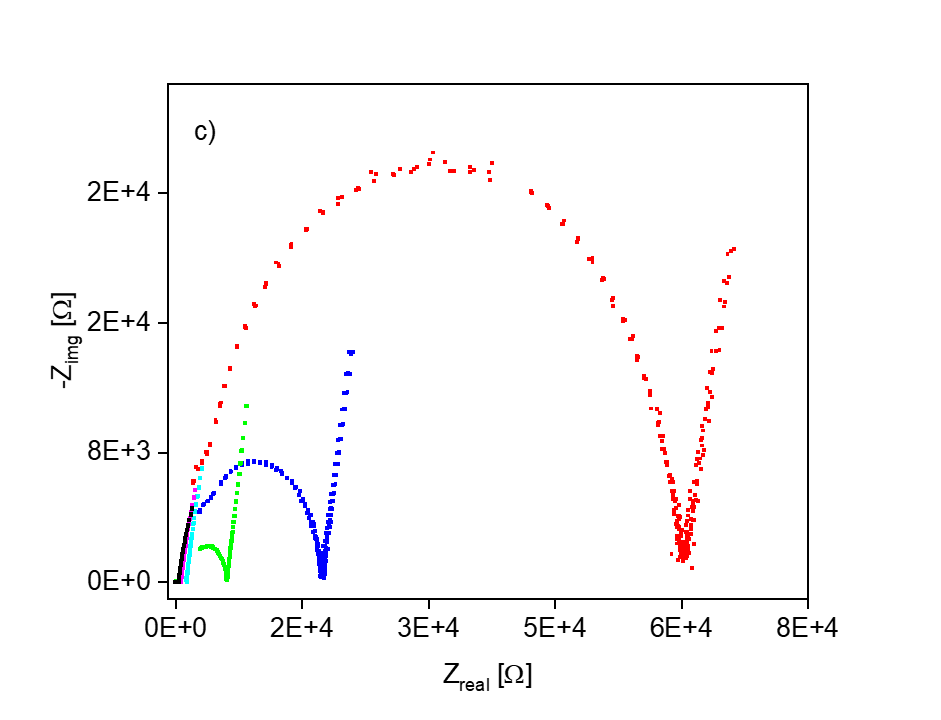** | **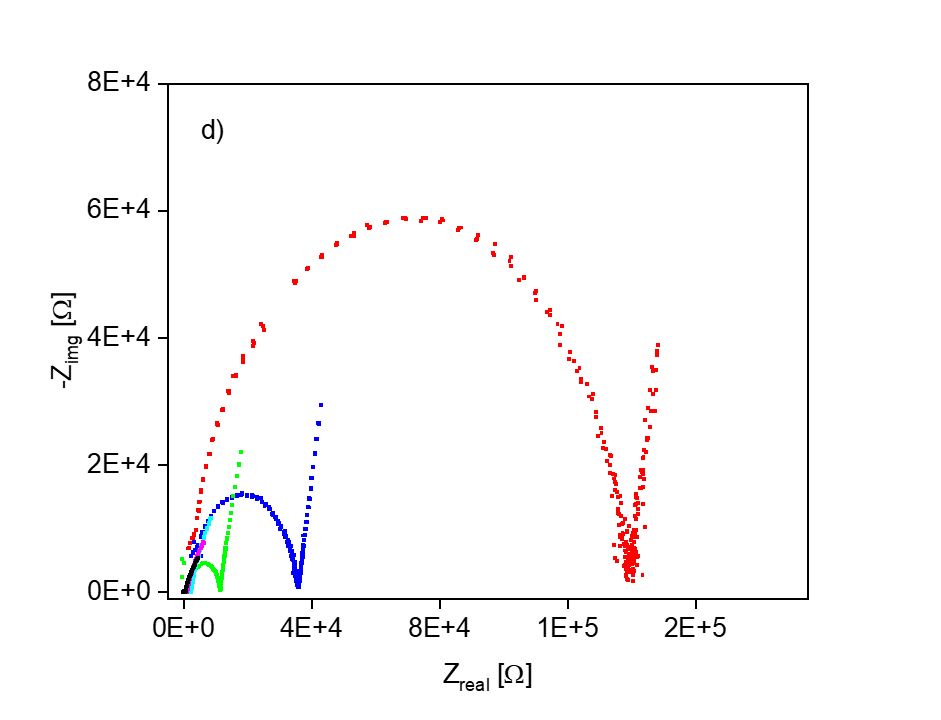** |

**Figure S8.** Nyquist plots of the temperature-depending EIS measurements of p(AAC6ImC6Cl)/ CL (mol%)/ conducting salt (mol%) IL networks: a) p(AAC6ImC6Cl)/2.5/0, b) p(AAC6ImC6Cl)/2.5/5, c) p(AAC6ImC6Cl)/5/0, d) p(AAC6ImC6Cl)/5/5 (red: 20 °C, blue: 30 °C, green: 40 °C, cyan: 50 °C, magenta: 60 °C, black: 70 °C).

| 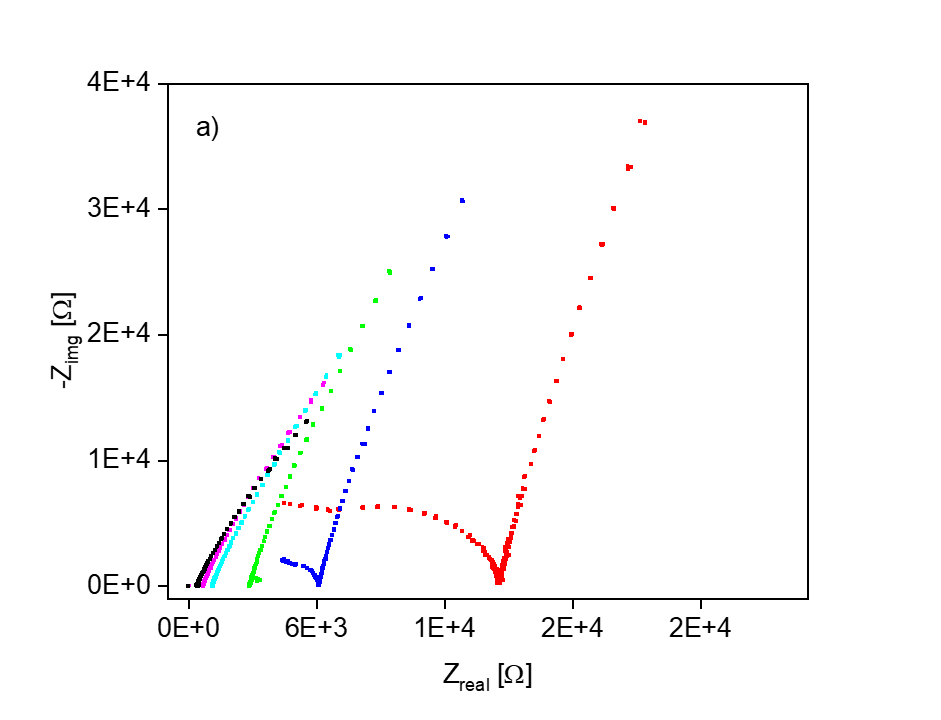 | **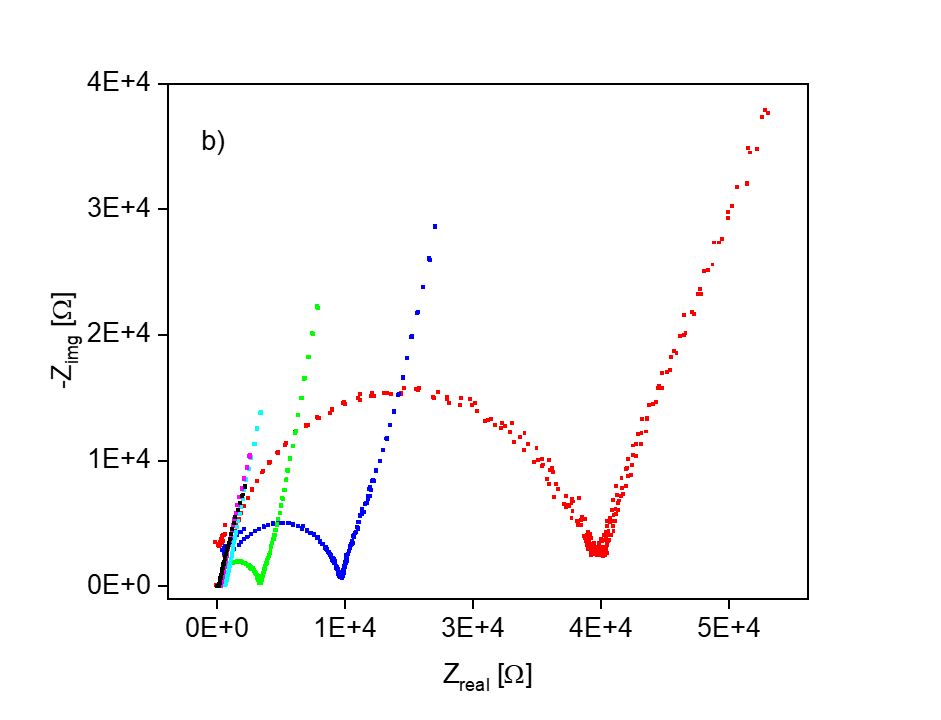** |
| --- | --- |
| **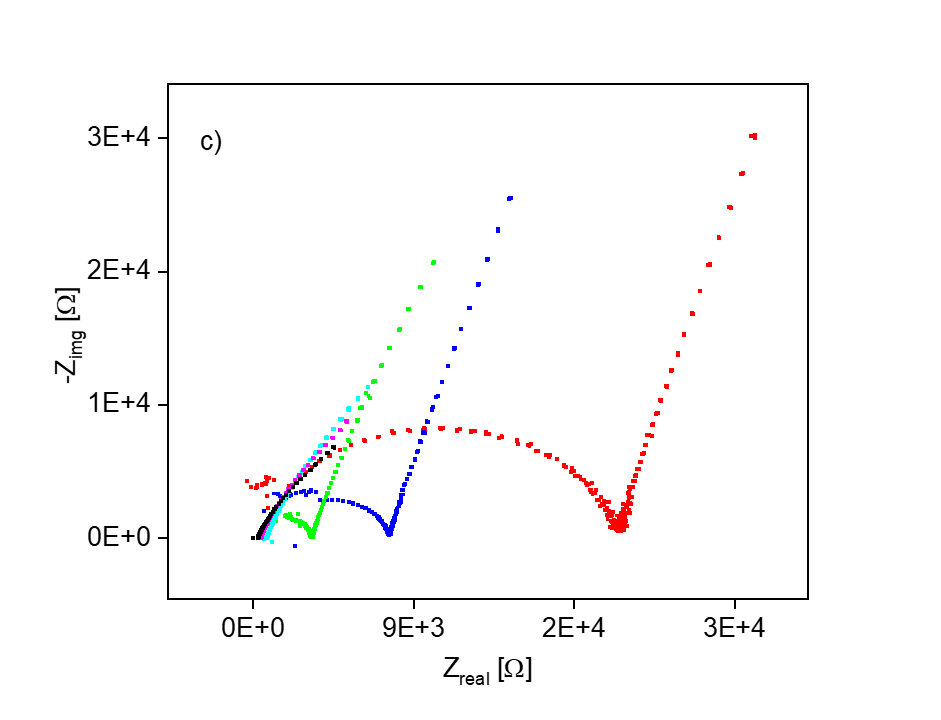** | |

**Figure S9.** Nyquist plots of the temperature-depending EIS measurements of p(AAC8ImC4Cl)/ CL (mol%)/ conducting salt (mol%) IL networks: a) p(AAC8ImC4Cl)/2.5/0, b) p(AAC8ImC4Cl)/5/0, c) p(AAC8ImC4Cl)/5/5 (red: 20 °C, blue: 30 °C, green: 40 °C, cyan: 50 °C, magenta: 60 °C, black: 70 °C).

1. **EIS spectra after swelling**


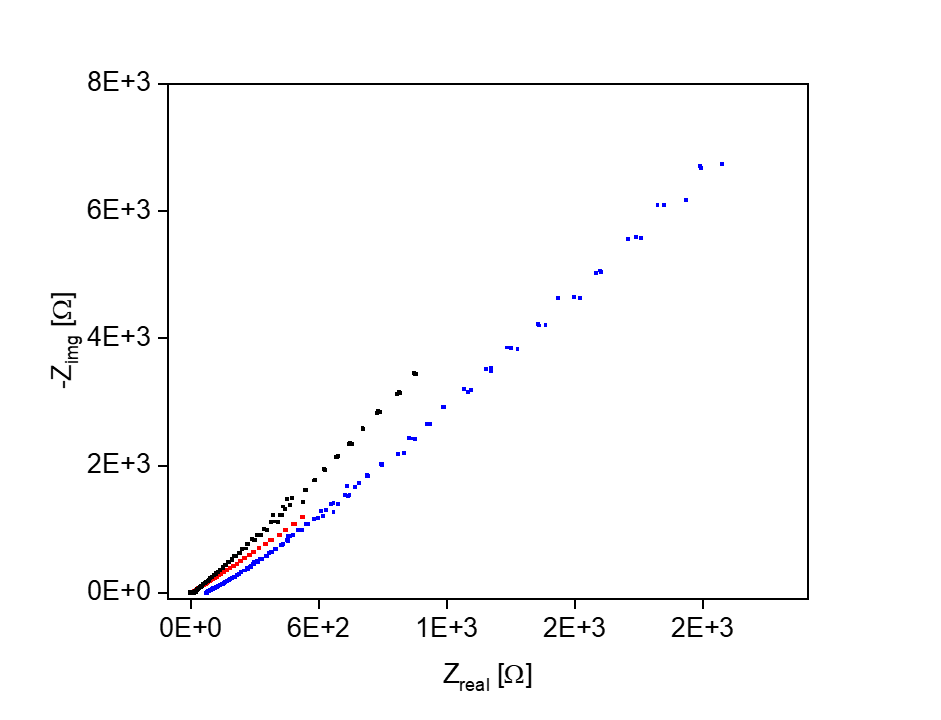


**Figure S10.** Nyquist plots of the p(AAC6ImC6Cl)/ CL (mol%)/ conducting salt (mol%) IL networks after swelling in water at 20 °C, red: p(AAC6ImC6Cl)/2.5/0, blue: p(AAC6ImC6Cl)/2.5/5, black: p(AAC6ImC6Cl/5/5).


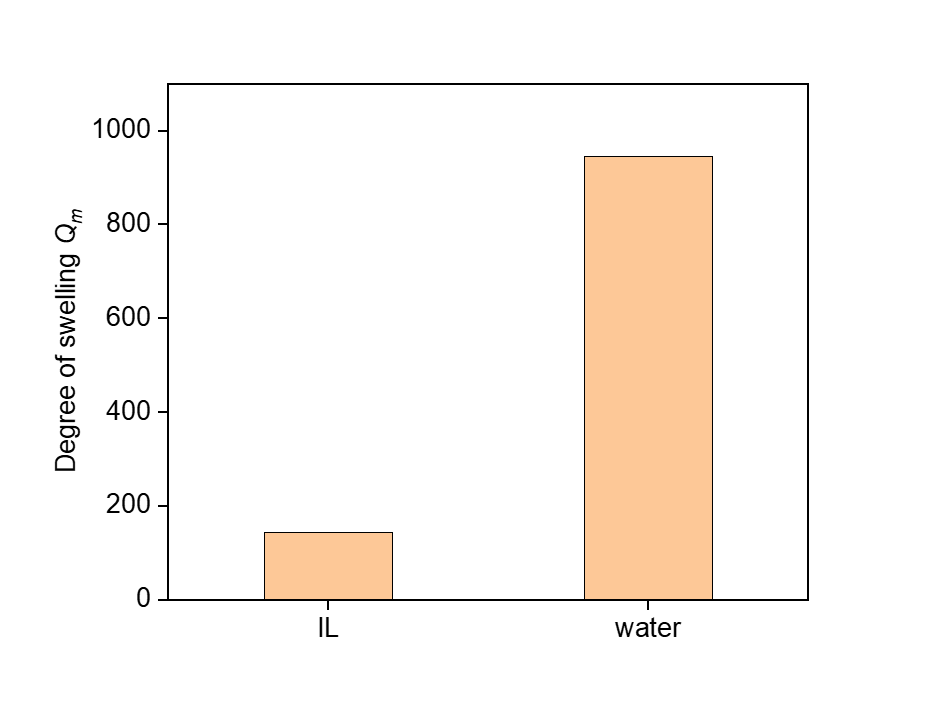


**Figure S11.** Swelling behavior of p(AAC6ImC4Cl)/5/5 in the ionic liquid 1-hexyl-3-methylimidazolium chloride and in water.

1. **Battery tests**

| **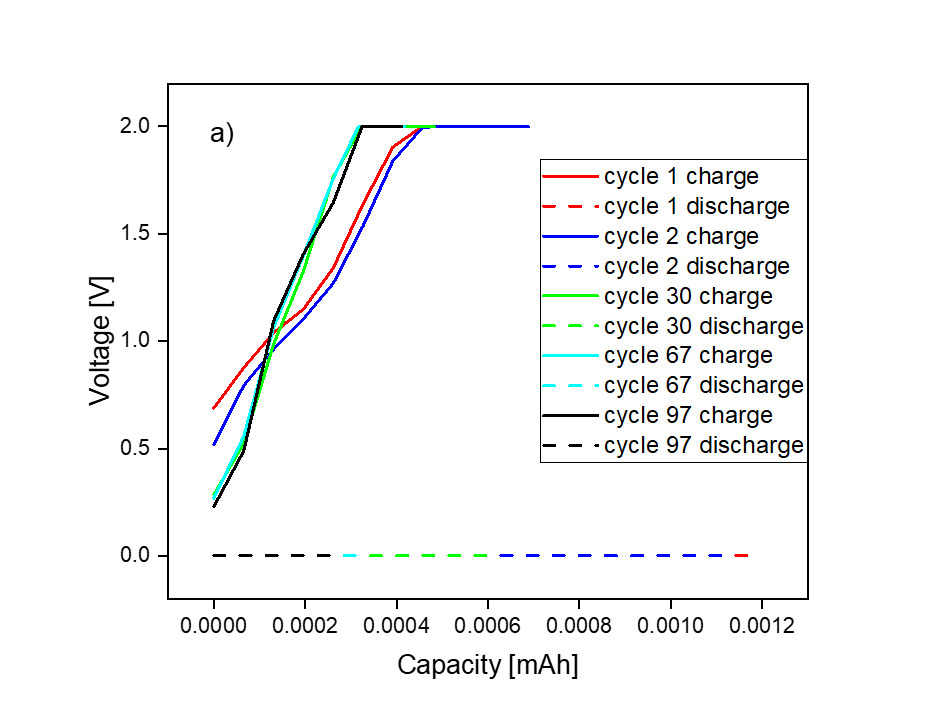** | **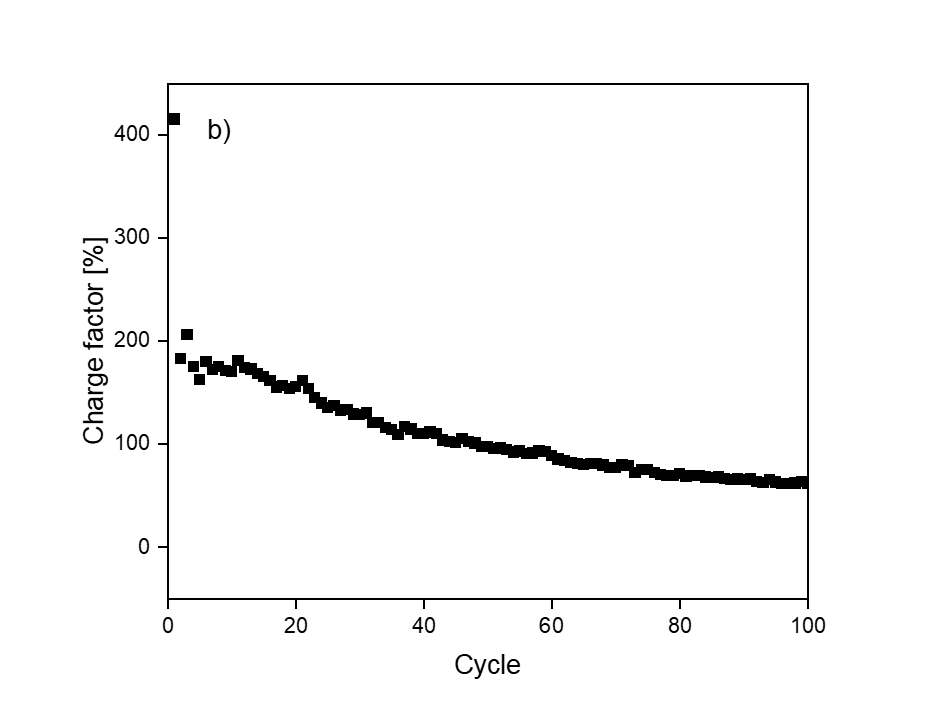** |
| --- | --- |

**Figure S12.** First test: **battery** p(TEMPO-MA)/zinc with PEL in solution (liquid electrolyte: 1M Zn(ClO_4_)_2_·6H_2_O in a mixture of EC/DMC in 1:1 ratio with linear homopolymer p(AAC6ImC4Cl)).

| **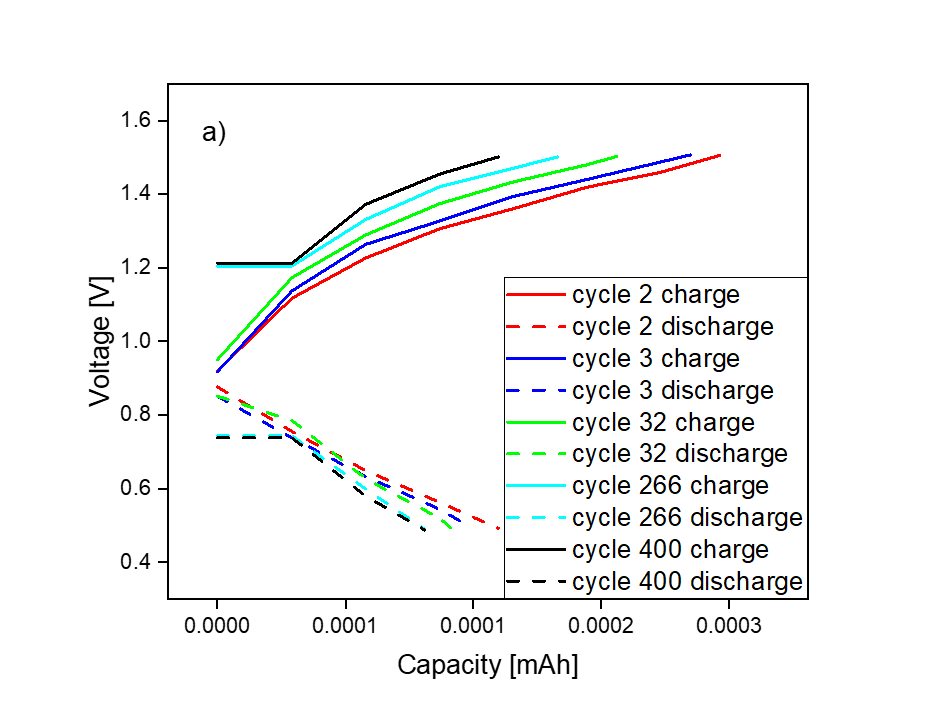** | **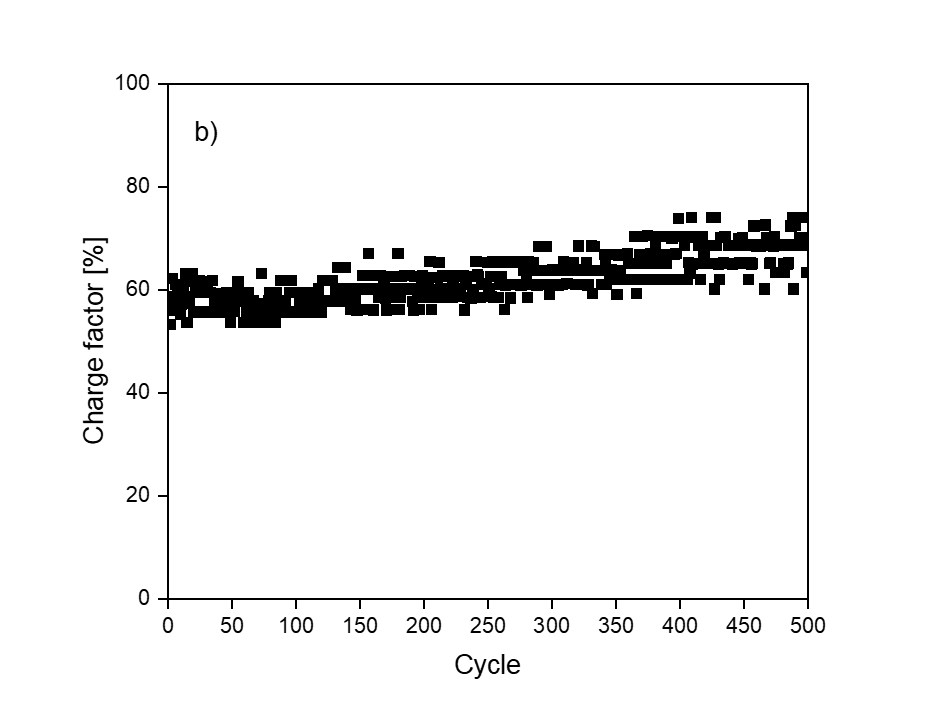** |
| --- | --- |

**Figure S13.** Second test: battery cell p(TEMPO-MA)/zinc with gel electrolyte: p(AAC6ImC4Cl)/5/5 with 20 wt% water.

**
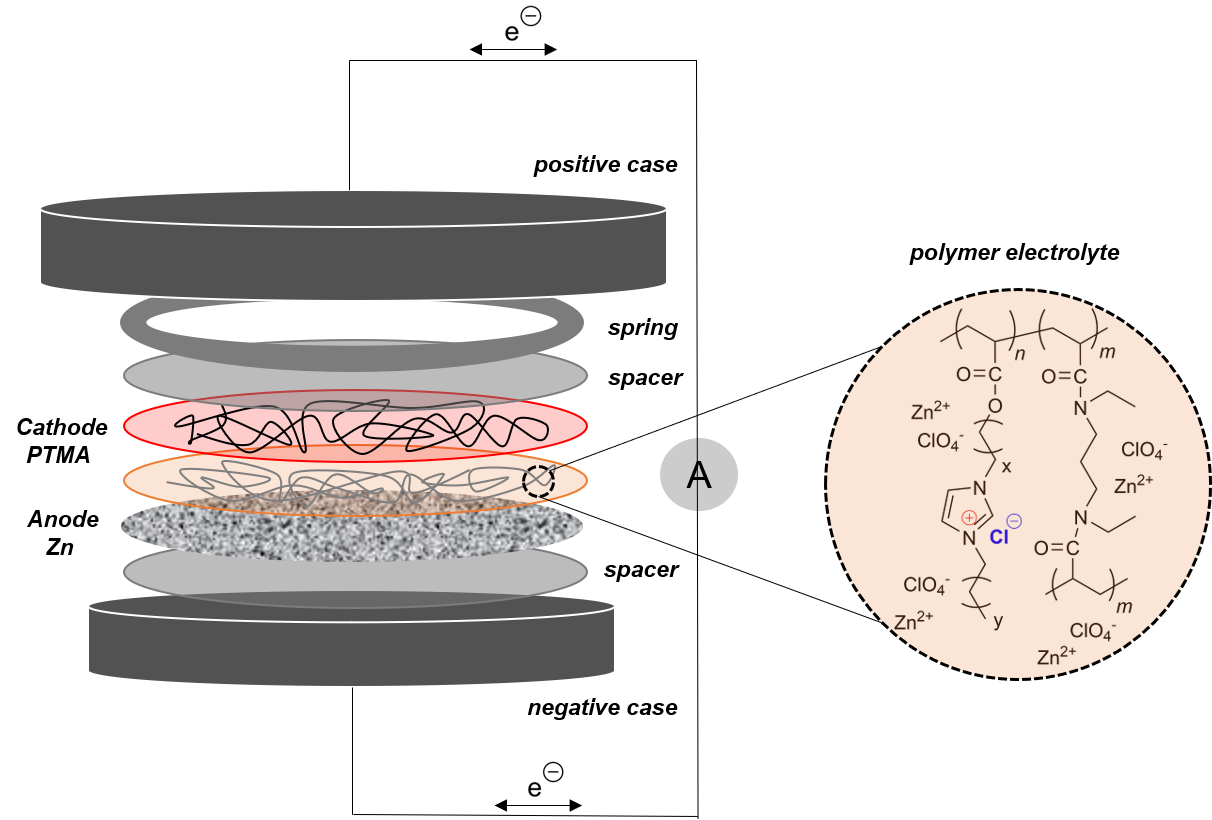
**

**Figure S14.** Setup of the p(TEMPO-MA)/zinc battery with PEL p(AAC6ImC4Cl)/5/5.

**
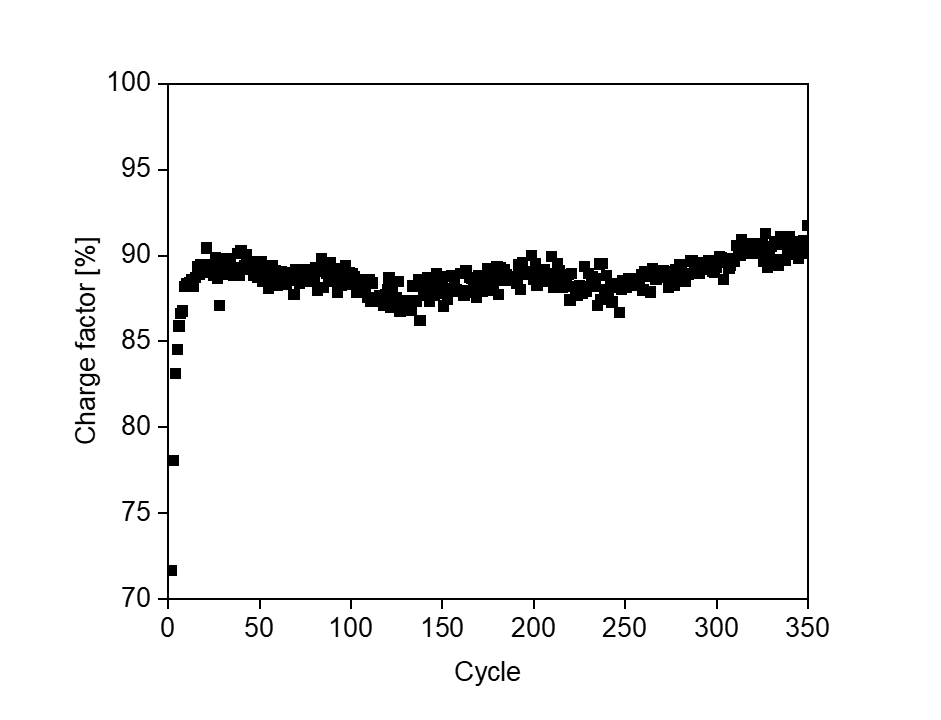
**

**Figure S15.** Charge factor of the p(TEMPO-MA)/zinc battery with polymer electrolyte during 350 cycles.
